# Convergent evolution of monocyte differentiation in adult skin instructs Langerhans cell identity

**DOI:** 10.1126/sciimmunol.adp0344

**Published:** 2024-09-06

**Authors:** Anna Appios, James Davies, Sofia Sirvent, Stephen Henderson, Sébastien Trzebanski, Johannes Schroth, Morven L. Law, Inês Boal Carvalho, Marlene Magalhaes Pinto, Cyril Carvalho, Howard Yuan-Hao Kan, Shreya Lovlekar, Christina Major, Andres Vallejo, Nigel J. Hall, Michael Ardern-Jones, Zhaoyuan Liu, Florent Ginhoux, Sian M. Henson, Rebecca Gentek, Elaine Emmerson, Steffen Jung, Marta E. Polak, Clare L. Bennett

**Affiliations:** 1 Department of Haematology, UCL Cancer Institute, University College London, London WC1E 6DD, UK; 2 Systems Immunology Group, Clinical and Experimental Sciences, Faculty of Medicine, University of Southampton, Southampton SO17 1BJ, UK; 3 Bill Lyons Informatics Centre, Cancer Institute, University College London, London WC1E 6DD, UK; 4 Department of Immunology and Regenerative Biology, Weizmann Institute of Science, Rehovot 76100, Israel; 5 William Harvey Research Institute, Barts & London School of Medicine and Dentistry, Queen Mary University of London, Charterhouse Square, London EC1M 6BQ, UK; 6 Centre for Reproductive Health, Institute for Regeneration and Repair, University of Edinburgh, Edinburgh, EH16 4UU, UK; 7 University Hospital Southampton NHS Foundation Trust, Southampton SO16 6YD, UK; 8 Human Development and Health, Faculty of Medicine, University of Southampton, Southampton SO17 1BJ, UK; 9 Dermatopharmacology, Clinical and Experimental Sciences, Faculty of Medicine, University of Southampton, Southampton So17 1BJ, UK; 10 Institute for Life Sciences, University of Southampton, Southampton SO17 1BJ, UK; 11 Shanghai Institute of Immunology, Department of Immunology and Microbiology, Shanghai Jiao Tong University School of Medicine, Shanghai 200025, China; 12 Singapore Immunology Network, Agency for Science, Technology and Research, Singapore 138648, Singapore; 13 Institut Gustave Roussy, INSERM U1015, Bâtiment de Médecine Moléculaire, Villejuif 94800, France; 14 Institute for Regeneration and Repair, University of Edinburgh, Edinburgh EH16 4UU, UK

## Abstract

Langerhans cells (LCs) are distinct among phagocytes, functioning both as embryo-derived, tissue-resident macrophages in skin innervation and repair and as migrating professional antigen-presenting cells, a function classically assigned to dendritic cells (DCs). Here, we demonstrate that both intrinsic and extrinsic factors imprint this dual identity. Using ablation of embryo-derived LCs in the murine adult skin and tracking differentiation of incoming monocyte-derived replacements, we found intrinsic intraepidermal heterogeneity. We observed that ontogenically distinct monocytes give rise to LCs. Within the epidermis, Jagged-dependent activation of Notch signaling, likely within the hair follicle niche, provided an initial site of LC commitment before metabolic adaptation and survival of monocyte-derived LCs. In the human skin, embryo-derived LCs in newborns retained transcriptional evidence of their macrophage origin, but this was superseded by DC-like immune modules after postnatal expansion. Thus, adaptation to adult skin niches replicates conditioning of LC at birth, permitting repair of the embryo-derived LC network.

## Introduction

Langerhans cells (LCs) are a specialized and highly conserved population of mononuclear phagocytes that reside in the outer epidermis of the skin. Initially defined as prototypic dendritic cells (DCs) because of their potential to migrate to draining lymph nodes (LNs) and initiate T cell immunity (1), subsequent fate-mapping studies supported a common origin with tissue macrophages in other organs (2). Hence, LCs are the only resident macrophage population that acquires the DC-like ability to migrate out of the tissue (3). Unlike DCs, however, LCs depend on colony-stimulating factor 1 receptor (CSF1R) signaling for survival (4) and perform more macrophage-like functions via interaction with peripheral nerves (5) and promotion of angiogenesis during wound healing (6). However, the signals that control this functional dichotomy within the spatial context of intact skin remain poorly defined.

Tissue-resident macrophage (TRM) identity is imprinted on fetal and adult monocyte precursors by the local anatomical niche wherein instructive signals permit convergent differentiation and survival of resident cells irrespective of ontogeny (7, 8). Environmental signals determine TRM identity via epigenetic regulation of specific transcription factor networks (9, 10) controlled by the transcription factor zinc finger E-box-binding homeobox 2 (Zeb2) (11, 12). These niches have been carefully delineated in the lungs and liver where interaction with local epithelia supports differentiation of TRM populations (13, 14). Implicit in these models is the concept of a single niche that provides a physical scaffold, trophic factors to support maintenance of the network, and the signals to imprint a TRM identity specific to that site (15). However, we questioned whether this model would also apply to LCs in the skin wherein monocytes exiting the blood must traverse the dermis and cross a basement membrane to (re)populate the LC network.

Murine CX3CR1^+^ fetal macrophage precursors enter the developing skin and differentiate into LC-like cells (2, 16) but do not mature into bona fide embryo-derived LCs (eLCs) until after birth (17, 18). By contrast, human eLCs differentiate within the epidermis before birth and histologically resemble adult cells by an estimated gestational age of 18 weeks (19, 20). Entry of LC precursors into the bone morphogenetic protein 7– and transforming growth factor–β (TGFβ)–rich environment of the epidermis results in activation of a runt-related transcription factor 3 (Runx3)– and inhibitor of DNA binding 2 (Id2)–dependent program of differentiation (2, 3, 21, 22), defined by expression of the c-type lectin Langerin (CD207) and high expression of cell adhesion molecules including epithelial cell adhesion molecule (EpCAM) and E-cadherin. Once resident, eLCs depend on interleukin-34 (IL-34) for survival (23, 24), and the network is maintained throughout life via local proliferation of mature LCs (25–27), independent of the adult circulation. In both mice and humans, eLCs are sparse at birth but undergo a proliferative burst within the first week of exposure to the external environment in mice (18) and within 2 years of age in humans (19), but we do not know whether or how this postnatal transition may shape eLC identity and function.

Pathological destruction of the eLC network during graft-versus-host disease (GVHD) results in replacement of eLCs with donor bone marrow (BM)–derived LCs (28, 29). We and others have shown that acute inflammation and destruction of eLCs in murine models of GVHD or ultraviolet irradiation triggers an influx of monocytes to the epidermis (4, 30–32). By tracking monocyte differentiation, we demonstrated that epidermal monocytes undergo differentiation to EpCAM^+^CD207^neg^ precursors, which become long-lived monocyte-derived LCs (mLCs) that are transcriptionally similar to the cells they replace, are radioresistant, and acquire DC-like functions of migration to LNs and priming of T cells (32). These data suggested that migration into the epidermal environment was sufficient to instruct differentiation of short-lived monocytes into long-lived LCs. However, lineage-tracing studies have revealed heterogeneity within classical Ly6C^+^ monocytes such that both granulocyte-macrophage progenitors (GMPs) and monocyte-DC progenitors (MDPs) can give rise to classical monocyte populations (33, 34) and differentially seed TRM populations across the body (35). These data suggested that intrinsic factors determined by monocyte ontogeny may also shape tissue macrophage differentiation.

Here, we sought to determine how intrinsic and extrinsic factors combine to direct monocyte differentiation in the epidermis and whether the local skin environment plays an instructive or permissive role in this process. Using our model of LC replacement, we determined the process by which distinct cellular niches in the skin epidermis permit differentiation and survival of long-lived resident LCs. We demonstrate that a combination of BM monocyte ontogeny and environmental signals provided by the adult hair follicle niche instruct programs of LC development toward DC-like cells that replicate postnatal LC maturation in the human skin. Together, these data reveal mechanisms of convergent adaptation to the epidermal niche that imprints the distinct LC identity in the skin.

## Results

### Single-cell transcriptomics reveals monocyte-derived cell heterogeneity in the inflamed epidermis

**Fig. 1:**
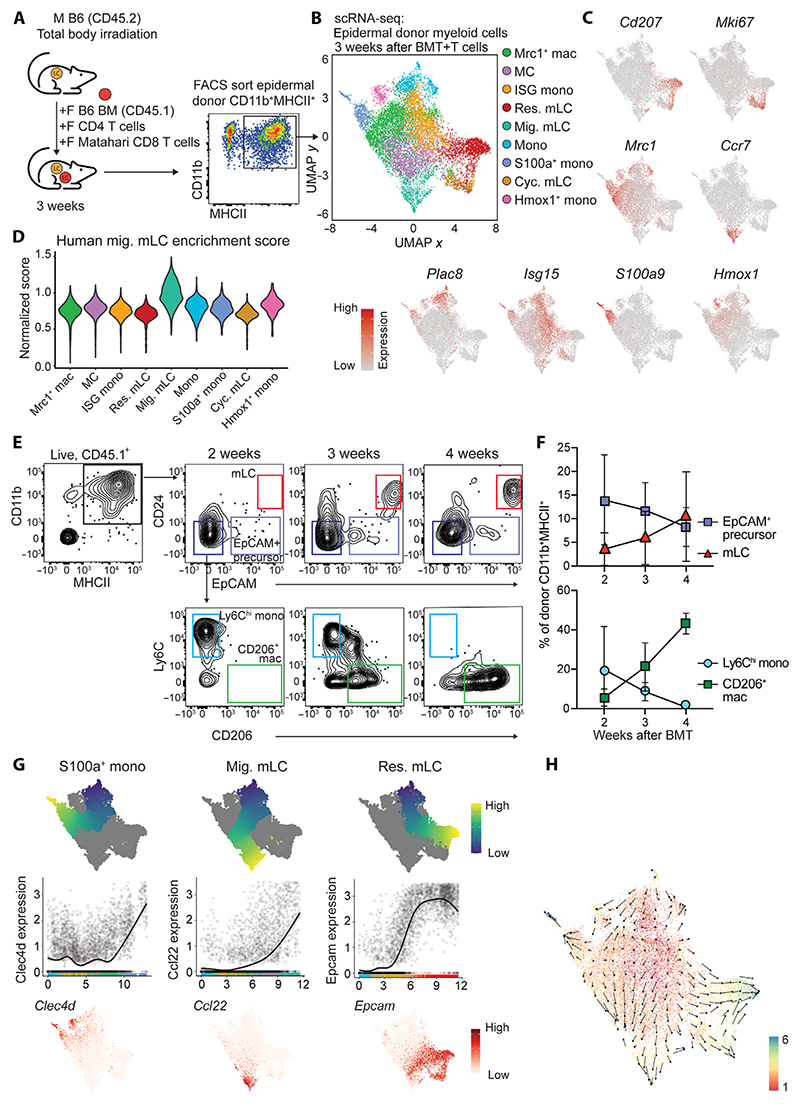
scRNA-seq reveals monocyte-derived cell heterogeneity in the inflamed epidermis. (**A**) Experimental design showing murine bone marrow transplant model and cells sorted for scRNA-seq from murine epidermis. M, male; F, female. For the full gating strategy, see fig. S1A. (**B**) Uniform Manifold Approximation and Projection (UMAP) and clustering of murine donor CD11b^+^MHCII^+^ cells from murine GVHD epidermis analyzed by scRNA-seq. Data are from two combined independent sorting and sequencing experiments using epidermis from 4 and 10 pooled mice. Mac, macrophage; res. mLC, resident mLC; mig. mLC, migratory mLC; mono, monocyte; cyc. mLC, cycling mLC. (**C**) Heatmap overlays showing expression of indicated genes across the dataset. Expression scales: *Cd207*, 0 to 4; *Mki67*, 0 to 4; *Mrc1*, 0 to 5; *Ccr7*, 0 to 4; *Plac8*, 0 to 5; *Isg15*, 0 to 4; *S100a9*, 0 to 6; *Hmox1*, 0 to 5. (**D**) Violin plot showing enrichment scores for a human mig. LC gene signature across clusters. (**E**) Representative flow plots showing donor CD11b^+^MHCII^+^ cells from murine GVHD epidermis at the indicated time points after BMT + T cells. Cells were pregated on live, singlet, CD45.1^+^ (donor) cells. (**F**) Quantification of populations indicated in (E). Data are presented as means ± S D, (*n* = 2 for 2 weeks, 8 for 3 weeks, and 2 for 4 weeks). Data are pooled from three independent experiments. (**G**) Differentiation trajectories calculated with Slingshot overlaid onto UMAP from (B) (above), normalized expression of indicated genes (*y* axis) across pseudo-time (*x* axis) for the indicated trajectories (middle), and feature plots showing normalized expression of indicated genes overlaid onto UMAP from (B) (below). Expression scales: *Clec4d*, 0 to 4; *Ccl22*, 0 to 6; *Epcam*, 0 to 4. (**H**) RNA velocity analysis applied to data from (B). Arrow directions indicate inferred cell trajectory.

To determine the molecular pathways that result in successful tissue residency and differentiation of mLCs in the adult skin, we exploited our murine model of minor h-antigen mismatched hematopoietic stem cell transplantation, in which allogeneic T cells destroy resident eLCs (26, 31). In this model, mLCs subsequently replace the eLC network (32). We carried out single-cell RNA sequencing (scRNA-seq) on sorted donor CD11b^+^ major histo-compatibility complex II (MHCII)^+^ cells isolated from the epidermis 3 weeks after BM transplant (BMT) with male antigen-specific Matahari T cells (after BMT + T cells) (Fig. 1A and fig. S1A) (36). Analysis at this time point allowed us to map the spectrum of CD11b^hi^ monocytes, CD11b^int^EpCAM^+^CD207^neg^ LC precursors, and CD11b^int^EpCAM^+^CD207^+^ LCs we have previously defined in the epidermis (32). Dimensionality reduction and clustering of the cells demonstrated unexpected heterogeneity within donor BM–derived cells, including several transcriptionally diverse clusters of cells that surrounded a central collection of still distinct but more convergent clusters (Fig. 1B). We used a parametric bootstrapping method, single-cell significance of hierarchical clustering, to demonstrate that the data, particularly the central clusters, were not overfit and thus likely to be biologically relevant (fig. S2A) (37).

Identification of the differentially expressed genes (DEGs) that defined the clusters (Fig. 1C and fig. S1B) revealed three populations of mLCs: resident mLCs (*Cd207, Epcam*, and *Mfge8*), cycling mLCs (*Top2a* and *Mki67*, which also retained weakened expression of the resident mLC signature), and cells that appeared to be poised for migration out of the epidermis, which we have termed migrating mLCs (*Cd83, Nr4a3*, and *Ccr7*) to reflect a similar term used for these cells in human LC datasets (38–40). Consistent with these human LC data, migrating mLCs down-regulated genes associated with LC identity (*Cd207* and *Epcam*) and, instead, expressed a generic monocyte-derived DC (moDC) signature (Fig. 1C and fig. S1B) that showed the highest enrichment score for human migrating LCs across all clusters (Fig. 1D) (38). These data suggest that some mLCs are constitutively primed for migration, as observed in human steady-state eLCs (39). We also identified a classical monocyte cluster that expressed *Plac8, Lyz2, Ly6c2, Tmbs10*, and *Chil3* (fig. S1B), likely to have recently arrived in the epidermis. These cells resembled monocytes recruited to skin wound sites early in the healing process (41). Two additional small populations of cells shared monocyte/neutrophil activation markers and signs of recent oxidative stress [S100 calcium binding protein (S100)a^+^ mono and heme oxygenase (Hmox)1^+^ mono] (Fig. 1C). The central overlapping clusters expressed genes associated with monocyte-derived macrophages in other tissues: The central cluster that shared most similarity to classical monocytes was defined as interferon-stimulated gene monocytes (ISG monos; *Isg15, Ifit2*, and *Ifit3*) because these genes were also evident in the classical monocyte cluster; conversely, we observed a cluster of *Mrc1* (encoding CD206)– and *Arg1*-expressing monocyte-derived macrophage-like cells (Mrc1^+^ macs) that resemble those found in the dermis (Fig. 1C and fig. S1B) (42). These cells appeared to have differentiated along a default macrophage pathway to express canonical tissue macrophage genes and shared some similarity to the converting macrophages recently identified in the pleural cavity after nematode infection (43). We also detected a separate cluster of cells that were identified by their up-regulation of *Ccl17, Mgl2, Dcstamp*, and *Itgax*, genes linked to moDCs (Fig. 1C and fig. S1B). Given the lack of clarity into the identity of these cells, we labeled them “converting monocyte-derived cells” (MCs), in reference to a similar transitional population of cells recently described (43). Flow cytometry data validated the heterogeneous cell fates, showing loss of monocytes over time with expansion of mLCs and CD206^+^ macrophages (Fig. 1, E and F). Analysis of eLCs coisolated from the GVHD skin at 3 weeks revealed three clusters of cells that resembled those identified in the human skin (39): Clusters 1 and 2 were defined as resident eLCs, whereas the third cluster mirrored *Ccl22*^+^*Nr4a3*^+^ migrating mLCs (fig. S2B).

To better understand the path by which monocytes became mLCs, we inferred the trajectory of monocyte development toward finite cell states using Slingshot and RNA velocity (Fig. 1, G and H) (44, 45). These analyses revealed S100a^+^ monocytes as one of the end-point cell states, illustrated by expression of *Clec4d* (Fig. 1G).

This Slingshot-derived pathway passed mainly via Mrc1^+^ macs, whereas RNA velocity also suggested that some of this may be because of direct differentiation of incoming monocytes via the *Hmox1*^+^ monocyte cluster (Fig. 1H). A separate pathway toward migratory mLC was inferred by steadily increasing expression of *Ccl22* (Fig. 1G); this distinct route is likely driven by the down-regulation of *Cd207*, which is not present in migratory mLCs (40). By comparison, up-regulation of *Epcam* defined monocytes destined to become resident mLCs (Fig. 1G). Although differentiation toward resident mLC populations was defined as an end point (Fig. 1H), the direction of differentiation predicted by RNA velocity displayed some uncertainty within Mrc1^+^ macs and ISG monos that was resolved once cells entered the MC cluster, congruent with expression of *Epcam* (Fig. 1G). This apparent lack of commitment was illustrated by the short latent time and relative increase in unspliced versus spliced transcripts within ISG monos and Mrc1^+^ macs compared with committed mLC populations (fig. S2C). Focusing on differentiation toward resident mLCs, progenitor marker gene analysis demonstrated loss of monocyte-specific genes (*Ly6c2* and *Plac8*) and acquisition of LC-defining genes (*Cd207, Epcam*, and *Mfge8*), as well as those associated with cell adhesion (*Cldn1*) and production of noninflammatory lipid mediators (*Ptgs1, Ltc4s, Lpar3*, and *Hpgds*) (fig. S2D).

Thus, specification of an mLC fate in the adult epidermis occurs in situ in the skin, but it is likely that not all monocytes receive these signals, and some undergo a default macrophage differentiation. Rather than discrete points of cell fate decisions, our combined analyses identify a continuum of gene expression across the central clusters that converge to program mLC development in some cells.

### Monocyte ontogeny determines mLC repopulation

**Fig. 2:**
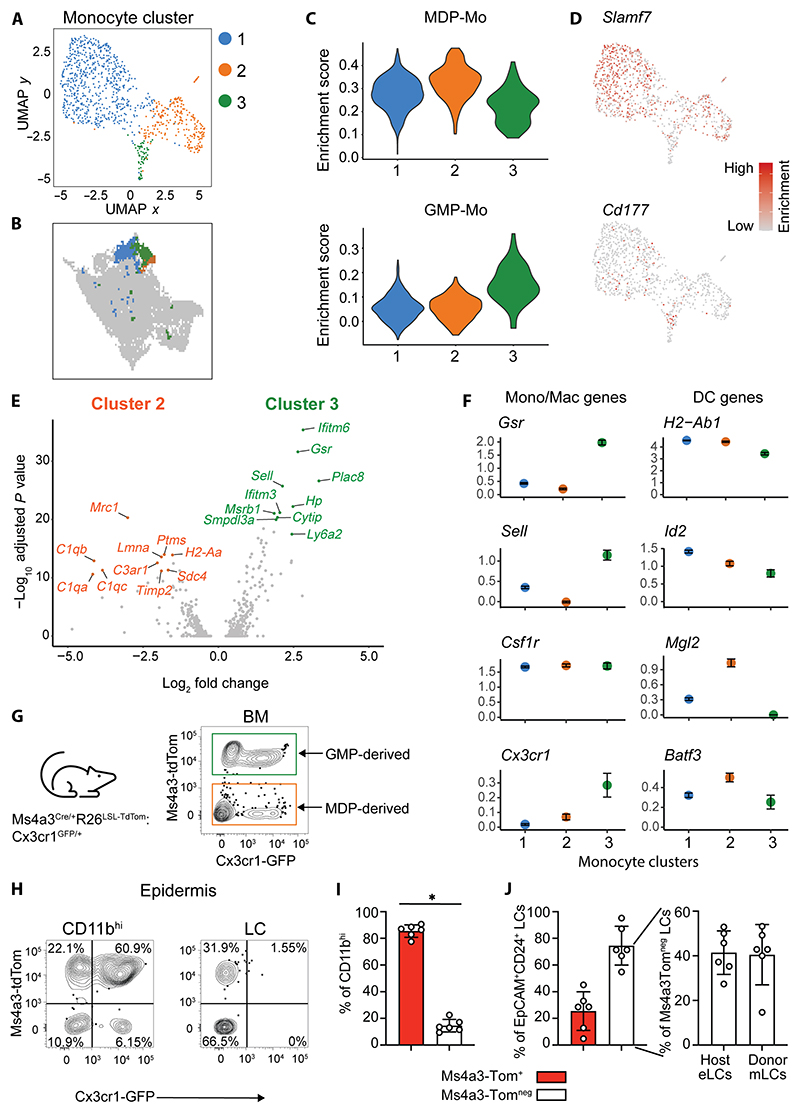
Monocyte ontogeny determines mLC repopulation. (**A**) UMAP and subclustering of monocytes from GVHD epidermis. (**B**) Clusters from (A) overlaid onto UMAP from Fig. 1B. (**C**) Violin plots showing enrichment scores for MDP-Mo (top) and GMP-Mo (bottom) gene signatures across clusters from (A). (**D**) Heatmap overlays showing normalized expression of indicated genes. Expression scales: *Slamf*7, 0 to 2; *Cd177*, 0 to 1.5. (**E**) Volcano plot showing DEGs between cluster 2 and cluster 3 from (A). The top 10 significant DEGs are highlighted. (**F**) Scatterplots of selected genes across monocyte clusters. (**G**) Schematic and representative flow plot showing Ms4a3-tdTom and Cx3cr1-GFP expression on live cells isolated from Ms4a3^Cre/+R26LSL-TdTomato^:Cx3cr1^GFP/+^ BM. (**H**) Representative contour plots showing Ms4a3-tdTom and Cx3cr1-GFP expression on epidermal CD11b^high^ monocytes and LCs 3 weeks after BMT + T cells. (**I**) Bar graph showing the frequency of GMP-derived (tdTom^+^) and MDP-derived (tdTom^−^) cells within epidermal CD11b^high^ cells. Data are represented as means ± SD (*n* = 6; ^*^P = 0.03, Wilcoxon matched pair test). (**J**) Left: Bar graph showing the frequency of tdTom^+^ and tdTom^−^ epidermal LCs. Right: Bar graph showing the frequency of host (CD45^+^) and donor (CD45^++^) cells within the tdTom^−^ EpCAM^+^CD24^+^ LC gate. Data are represented as means ± SD (*n* = 6). Data were pooled from two independent experiments.

Heterogeneity in epidermal monocyte fate may be explained by intrinsic bias within incoming Ly6C^hi^ monocytes and/or extrinsic programming within a specified tissue niche. To test the first possibility, we investigated the epidermal monocyte population in more detail. Reclustering of the classical monocyte cluster revealed three clusters of cells that were also evident within the parental CD11b^+^MHCII^+^ cell dataset (Fig. 2, A and B). Enrichment of gene signatures for GMP-derived monocytes (GMP-Mos) and MDP-derived monocytes (MDP-Mos) (35) and expression of the defining genes *Cd177* and *Slamf7* suggested that cluster 3 represented GMP-Mos, whereas cluster 2 was MDP-Mos (Fig. 2, C and D). This identification was supported by direct comparison between clusters 2 and 3; cluster 2 cells expressed higher levels of *Sell* (encoding CD62L) and classical monocyte genes (*Plac8* and *Ly6a2*), whereas cluster 3 expressed *C1q* genes and *H2-Aa*, which are associated with MDP-Mos (Fig. 2E). To better understand the macrophage/DC potential within monocyte subsets, we compared expression of a defined panel of genes associated with each cell type: cluster 3 (GMP-Mos) appeared more macrophage-like with higher expression of the glutathione reductase (*Gsr*) and *Cx3cr1*; cluster 2 was distinguished by increased, but differential, expression of *Id2, Mgl2*, and *Batf3*, suggesting a closer relationship with DC-like cells (Fig. 2F and fig. S3A). *Mgl2* and *Ccl17*, markers of MDP-Mo progeny in the lungs (35), were expressed within MDP-Mo clusters (Fig. 2F and fig. S3A) and also within the MC cluster from our scRNA-seq dataset (fig. S1B). Cluster 1 was the dominant population that appeared to bridge clusters 2 and 3, contained the bulk of differentiated cells when considered in the context of the complete dataset (Fig. 2B), and had down-regulated genes expressed by MDP-Mo/cluster 2 while acquiring expression of GMP-Mo–associated genes such as *Sell* (Fig. 2F), but maintained high expression of the LC-defining transcription factor *Id2*. Therefore, it was possible that these cells represented a mix of monocytes differentiating from clusters 2 and 3.

**Fig. 3:**
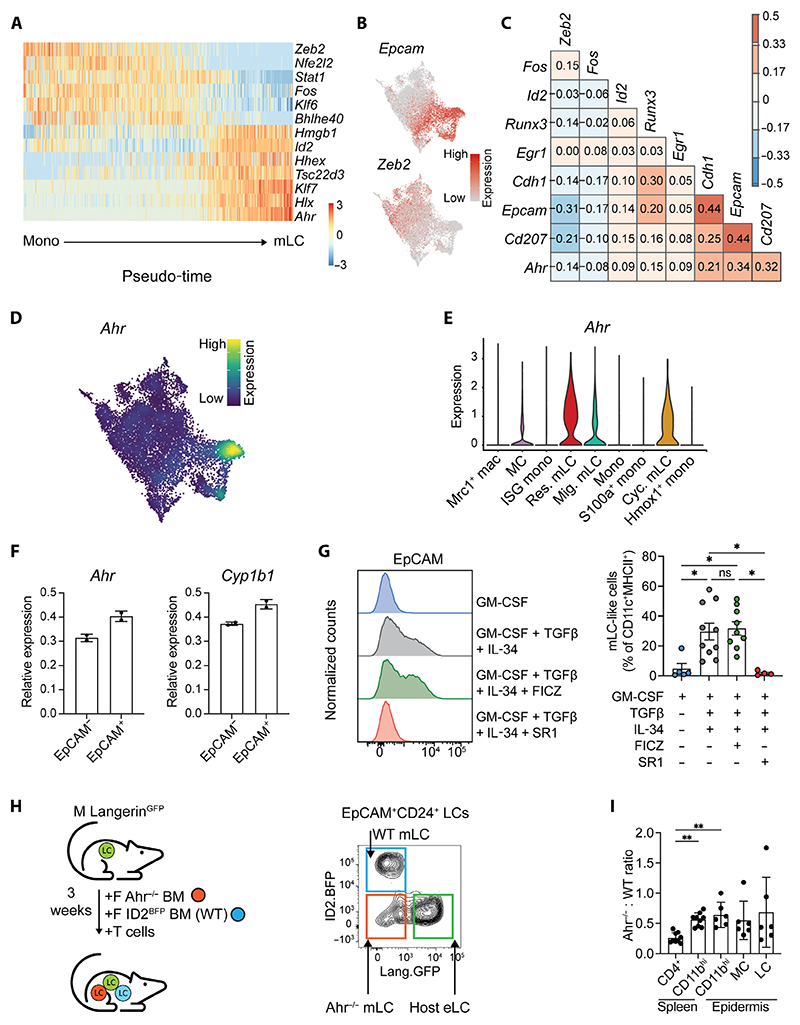
mLC differentiation is associated with loss of Zeb2 and up-regulation of Ahr. (**A**) Heatmap showing scaled gene expression of transcription factors that are differentially expressed along the differentiation trajectory (pseudotime) from monocyte to res. mLC. (**B**) Heatmap overlays showing normalized expression of indicated genes across UMAP from Fig. 1B. Expression scales: *Epcam*, 0 to 4; *Zeb2*, 0 to 3. (**C**) Correlation of selected LC-defining genes (*y* axis) across all clusters of the scRNA-seq dataset. (**D**) Density plot showing expression *Ahr* across cells from scRNA-seq dataset; expression scale from 0 to 0.06. (**E**) Violin plot showing normalized expression of *Ahr* across clusters from the scRNA-seq dataset. (**F**) Bar graphs showing the relative expression (means ± SD) of *Ahr* and *Cyp1b1* in sorted CD11b^+^EpCAM^neg^ and CD11b^+^EpCAM^+^ cells generated in vitro in the presence of FICZ. Expression is normalized to cells treated with GM-CSF + TGFβ + IL-34 alone (*n* = 2 independent experiments). (**G**) Representative histogram overlay (left) of EpCAM expression by monocytes cultured for 6 days under the indicated conditions and summary bar graph (right) of mLC-like cells generated from these conditions (for gating, see fig. S4D). Data are represented as means ± SD (*n* = 5 independent experiments). Statistical differences were assessed using Kruskal-Wallis with Dunn’s multiple comparison test, ^*^P < 0.05. ns, not significant. (**H**) Left: Schematic showing the experimental setup to generate competitive chimeras. Male Langerin^DTR.GFP^.B6 mice received a 1:1 mix of BM from female Ahr-replete [Ahr^+/+^.Id2^BFP^.B6 reporter mice, wild-type (WT)] or Ahr-deficient (Ahr^−/−^.B6) donors with Matahari T cells, and donor chimerism was assessed in the epidermis and spleen 3 weeks after transplant. Right: Representative contour plot showing gating of the different populations in the epidermis. (**I**) Bar graph showing ratio of Ahr^−/−^ to WT frequencies of indicated cell types in the spleens and epidermis of transplanted mice. Data are represented as means ± SD (*n* = 6 for epidermis and 9 for spleens, from two or three independent experiments). Significant differences were assessed using Kruskal-Wallis with Dunn’s multiple comparison test, ^**^P < 0.01.

To test whether different monocytic precursors were intrinsically biased toward becoming mLCs, we first sorted GMPs, MDPs, or total Ly6C^high^ monocytes from murine BM (gating strategy in fig. S3B) and cultured these cells with granulocyte-macrophage colony-stimulating factor (GM-CSF), TGFβ, and IL-34 to promote generation of CD24^+^EpCAM^+^ mLC-like cells (21, 32, 46). MDPs, but not GMPs, generated mLCs in vitro (fig. S3, C and D). However, total Ly6C^high^ monocytes were consistently superior at generating CD24^+^EpCAM^+^ mLC-like cells (fig. S3, C and D). Therefore, we exploited recently described Ms4a3^Cre/+^:R26^LSL-TdTomato^:Cx3cr1^GFP/+^ lineage reporter mice to track the fate of GMP-Mos in vivo (35). These mice permitted tracing of GMP progeny via *Ms4a3*-dependent tdTomato (tdTom) expression (47), with green fluorescent protein (GFP) labeling of BM and blood monocytes, but not LCs (48). BM cells from these mice demonstrated the expected expression pattern of fluorescent proteins (Fig. 2G). Injection of BM from female Ms4a3^Cre/+^:R26^LSL-TdTomato^:Cx3cr1^GFP/+^ mice into male wild-type recipients resulted in the expansion of both tdTom^+^GFP^+^ and tdTom^neg^GFP^+^ CD11b^high^ donor cells in the epidermis 3 weeks after transplant (Fig. 2, H and I), supporting our scRNA-seq data showing the recruitment of both GMP-derived (tdTom^+^) and MDP-derived (tdTom^neg^) monocytes. Some of these cells had already begun to down-regulate expression of Cx3cr1/GFP, consistent with the loss of *Cx3cr1* in our cluster 1 cells (Fig. 2F), before becoming CX3C1^neg^ LCs (Fig. 2H). However, the contribution of GMP-Mos was more substantial than predicted, representing 85.5% ± 1.9 (SEM, *n* = 6) of the total population of donor CD11b^high^ cells (Fig. 2I). We were precluded from using congenic hosts in these experiments because of rejection of the donor BM from our CD45.1/B6 recipients, and Ms4a3-tdTom^neg^ LCs contained both MDP-Mo–derived mLCs and residual eLCs at this time point. To distinguish the resident and recruited (donor) LC populations, we took advantage of an observation that eLCs expressed lower levels of CD45 than mLCs (fig. S3, E and F). Most Ms4a3-tdTom^neg^ LCs were host eLCs at this time point, but Ms4a3-tdTom^neg^ donor mLCs contributed to this population (Fig. 3J and fig. S3G). Thus, despite the relative infrequency of MDP-Mos in the CD11b^high^ population, both GMP- and MDP-derived cells gave rise to committed EpCAM^+^CD24^+^ mLCs, which had down-regulated expression of Cx3Cr1 (Fig. 2, H and J). Together, these data suggest that damage to the eLC network initiates recruitment of both GMP-Mos and MDP-Mos, which expand via a common intermediary to reestablish the nascent mLC network.

### Differentiating monocytes lose *Zeb2*-regulated macrophage identity to become mLCs independent of aryl hydrocarbon receptor signaling

Analysis of the transcription factors that showed the closest correlation with the Slingshot trajectory from monocytes to resident mLCs revealed that the predicted differentiation trajectory was dominated by loss of the tissue macrophage–specifying factor Zeb2 (Fig. 3A). Expression of *Zeb2* was mutually exclusive to *Epcam*^+^ cells (Fig. 3, B and C), suggesting that, unlike other resident macrophage populations, *Zeb2* expression is suppressed during specification of mLCs.

Epidermal monocytes also down-regulated expression of Krüppel-like factor 6 (*Klf6*), which has been linked to proinflammatory gene expression, and loss of which is therefore consistent with emergence of quiescent LCs (49), as well as the transcription factors *Fos* and *Stat1. Zeb2* was highly negatively correlated with expression of the LC-defining transcription factor *Id2* and the aryl hydrocarbon receptor (*Ahr*) that are also up-regulated by eLCs in utero (Fig. 3, A to C) (2). Mirroring DCs that migrate out of the skin, mLCs poised for migration were defined by up-regulation of *Irf4, Rel*, and *Nr4a3* (fig. S4A) (38, 40, 50, 51).

Previous work has shown that monocyte expression of Ahr biases differentiation toward moDCs rather than monocyte-derived macrophages (52). Ahr is not required for eLC development from premacrophages in utero (fig. S4, B and C) (53), but we questioned whether Ahr signaling could be important for monocyte differentiation to mLCs in adult skin. Supporting this, *Ahr* was expressed by a small number of cells in the MC cluster, potentially indicating that those cells were en route to becoming mLCs (Fig. 3, D and E). To test whether Ahr signaling was required for monocyte differentiation in vitro, we generated mLCs in the presence or absence of the Ahr inhibitor StemRegenin1 or the agonist 6-formylindolo[3,2-b] carbazole (FICZ) (52). EpCAM^+^ cells were more sensitive to FICZ, which induced higher expression of *Ahr*, and the cytochrome P450 enzyme *Cyp1b1*, which is directly regulated by Ahr, compared with EpCAM^neg^ cells (Fig. 3F). However this activation of Ahr signaling did not result in an increase in the frequency of EpCAM^+^ mLC-like cells, probably because of Ahr ligands already present in culture media, such as tryptophan (Fig. 3G and fig. S4D) (54). By contrast, inhibition of Ahr signaling ablated differentiation of EpCAM^+^ cells, demonstrating a requirement for mLC development in vitro (Fig. 3G and fig. S4D). Guided by these data, we tested the requirement of mLCs on Ahr in vivo. Exploiting the expression of ID2 and Langerin by LCs, we used Id2^BFP^ (fig. S4, E and F) (55) reporter mice to generate competitive chimeras in which irradiated Langerin^GFP^.B6 males received a 1:1 mix of BM from female Ahr-replete (Ahr^+/+^. Id2^BFP^.B6) reporter mice or Ahr-deficient (Ahr^−/−^.B6) donors with Matahari T cells (Fig. 3H). Three weeks later, the epidermis was analyzed for presence of mLCs and precursor populations. Consistent with the requirement for Ahr in CD4^+^ T cells, Ahr-deficient BM cells did not contribute to repopulating splenic CD4^+^ T cells in chimeras (Fig. 3I) (53). We observed a slight bias toward Ahr-competent CD11b^+^ cells in the spleen across experiments (Fig. 3I), suggesting a systemic disadvantage toward loss of Ahr signaling; however, this ratio of Ahr-deficient to Ahr-replete cells was maintained and did not decrease within epidermal CD11b^+^ cells or their descendants (Fig. 3I). Therefore, these data suggested that Ahr signaling was not required for mLC differentiation in vivo. From these data, we proposed that loss of *Zeb2* expression is a critical step for differentiation of mLCs, and, although Ahr signaling was required for monocyte differentiation in vitro, regulation by Ahr did not determine an mLC fate within the adult skin.

### A distinct follicular keratinocyte niche imprints mLC fate

**Fig. 4:**
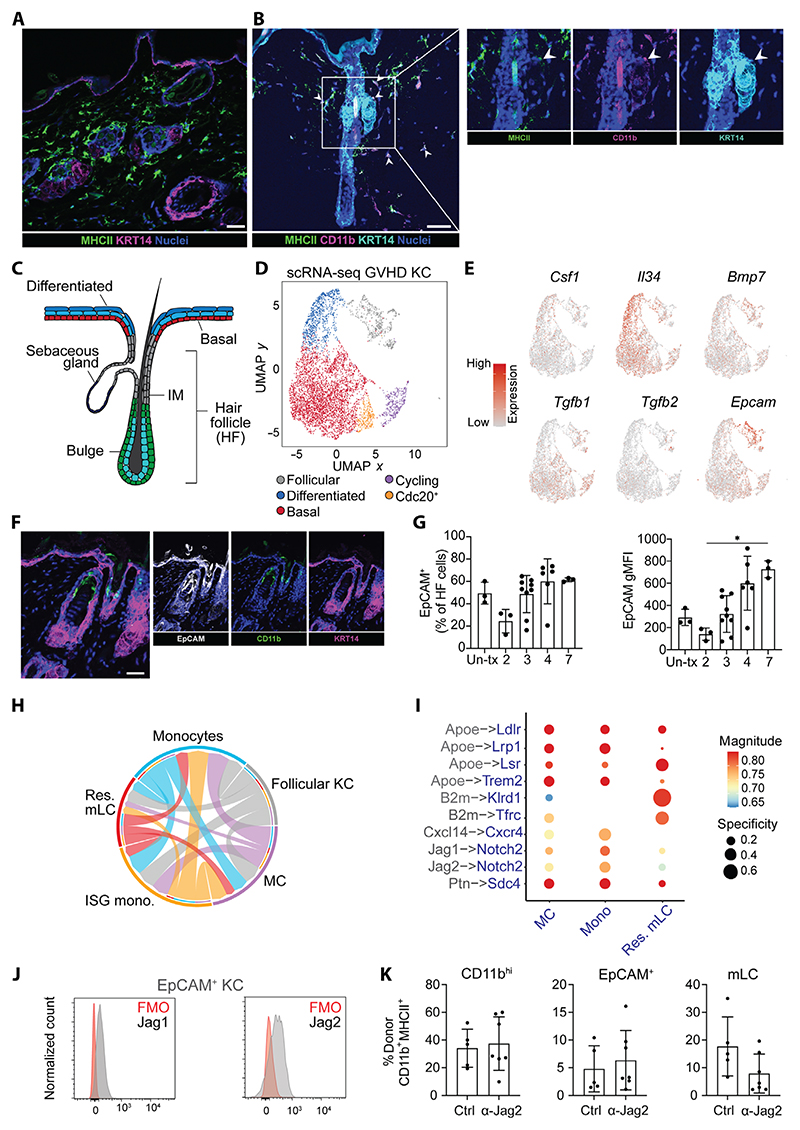
A specialized follicular keratinocyte niche imprints mLC fate. (**A**) Immunofluorescence (IF) image of murine epidermis 4 weeks after BMT + T cells: MHCII^+^ cells (green), KRT14^+^ keratinocytes (magenta), and nuclei (blue). Scale bar, 20 μm. (**B**) IF merged and single images of murine epidermis highlighting CD11b^+^MHCII^+^ cells at a KRT14^+^ upper hair follicle. Scale bar, 50 μm. (**C**) Schematic of murine hair follicle. IM, isthmus. (**D**) UMAP visualization of keratinocytes 3 weeks after BMT + T cells analyzed by scRNA-seq. Data were from epidermal cells of five pooled mice 3 weeks after BMT+ T cells. KC, keratinocytes. (**E**) Heatmap overlays showing normalized expression of indicated genes overlaid onto UMAP from (D). Expression scales: *Csf1*, 0 to 1.5; *Il34*, 0 to 2; *Bmp7*, 0 to 2.5; *Tgfb1*, 0 to 2; *Tgfb2*, 0 to 2; *Epcam*, 0 to 3. (**F**) Merged and single IF images of murine epidermis 4 weeks after BMT + T cells: EpCAM (white), CD11b (green), and KRT14 (magenta). Scale bar, 20 μm. (**G**) Bar graphs showing frequency and geometric mean fluorescent intensity (gMFI) of EpCAM^+^-expressing hair follicle cells from untransplanted (Un-tx) mice or after BMT + T cells. Data are means ± SD (*n* = 3 control; 2 weeks, *n* = 3; 3 weeks, *n* = 9; 4 weeks, *n* = 7; 7 weeks, *n* = 3), pooled from three independent experiments. Significance was calculated using Kruskal-Wallis with Dunn’s multiple comparison test, ^*^P < 0.05. (**H**) Chord plot showing receptor-ligand interactions between follicular KC (gray) and monocytes (blue), ISG monos (orange), MC (purple), and res.mLC (red) assessed by LIANA. The width/weight of each arrow indicates the number of potential interactions identified. (**I**) Dot plot showing the specificity (NATMI edge specificity) and magnitude (sca LR score) of interactions between follicular KC (gray) and indicated populations (blue). (**J**) Representative histograms of Jag1 and Jag2 expression by EpCAM^+^ KC in the epidermis. FMO, fluorescence minus one. (**K**) Bar graphs showing frequency of CD11b^high^, EpCAM^+^ precursors and mLCs in mice treated with anti–Jag2 antibodies or anti–IgG isotype control (Ctrl). Data are shown as means ± SD (control, *n* = 4; anti-Jag2, *n* = 7) and were pooled from two independent experiments.

To understand the signals regulating the transition from loss of a *Zeb2*-linked macrophage program to commitment to an LC identity, we next sought to define the mLC niche in vivo. Imaging of the skin after BMT + T cells revealed abundant MHCII^+^ cells in the inflamed dermis 3 weeks after transplant, with localization of CD11b^+^MHCII^+^ cells at the upper hair follicle epidermis, an anatomical site previously associated with monocyte recruitment to the epidermis (Fig. 4, A and B) (56). Therefore, to define where and how monocytes differentiated within potential epidermal niches, we performed scRNA-seq on CD45^neg^ keratinocytes (fig. S5A) and CD11b^+^MHCII^+^ cells sorted from the same epidermal samples 3 weeks after BMT + T cells and integrated the data with our existing CD11b^+^MHCII^+^ epidermal dataset. Clustering of CD45^neg^ cells followed by differential expression testing identified a set of cluster-specific markers that corresponded with cluster markers of a previously published mouse scRNA-seq dataset (57). The comparison indicated clusters of interfollicular epidermis–derived basal cells (*Krt14*^*high*^*Krt5*^*high*^) and terminally differentiated epidermal cells of the stratum spinosum (*Krt10*^*high*^), as well as a cluster that combined *Krt79*^*high*^*Krt17*^*high*^ cells of the upper hair follicle with a small subcluster of *Mgst1*^*+*^ cells that likely came from the sebaceous gland (Fig. 4, C and D, and fig. S5B). Two other clusters were identified as cycling cells (*Mki67*) and putative *Cdc20*^*+*^ stem cells.

To predict which keratinocytes could support differentiation of epidermal monocytes, we analyzed expression of factors known to be required for monocyte or mLC survival (*Csf1, Il34*, and *Bmp7*) and residency (*Tgfb1, Tgfb2*, and *Epcam*) (3). Although *Csf1* was not expressed by epidermal keratinocytes, *Il34*, which also binds the CSF1 receptor, was localized to *Krt10*^*high*^ terminally differentiated cells, consistent with its role as a survival factor for the mature LC network within the interfollicular epidermis (Fig. 4E). We could only detect low levels of *Tgfb1, Tgfb2*, and *Bmp7* transcripts, although *Tgfb1* was abundantly expressed across our epidermal myeloid cell dataset, consistent with its cell-autonomous function (fig. S5C) (58). By contrast, the TGFβ-activating integrin *Itgb8* (but not *Itgb6*) was specifically expressed by *Krt79*^*high*^ *Krt17*^*high*^ upper hair follicle cells as previously shown (fig. S5D) (59). We also detected highly specific restriction of *Epcam* by upper hair follicle cells (Fig. 4E). Expression of the cell adhesion molecule EpCAM is associated with residency of monocytes within the alveolar space and differentiation to alveolar macrophages (14), and EpCAM expression demarcates isthmus region epithelial cells of the upper hair follicle, which express chemokine (C-C motif) ligand 2 (CCL2) (56). Therefore, we postulated that this localized area may provide a niche for recruited monocyte-derived EpCAM^+^ LC precursors. CD11b^+^ monocytes were located at EpCAM-rich sites within the hair follicle (Fig. 4F). Protein analysis demonstrated high levels of EpCAM on follicular epithelium, which transiently decreased at the peak of T cell–mediated pathology in this model (Fig. 4G and fig. S5E) (31, 32), suggesting that loss of adhesion to this niche could contribute to the bottleneck in mLC differentiation that we observed in our previous study (32).

Using the ligand-receptor analysis framework (LIANA) (60), we predicted potential interactions between *Krt79*^*high*^*Krt17*^*high*^ follicular clusters and clusters that lay along the increasing EpCAM expression axis (monocytes, ISG monos, MCs, and resident mLCs). This analysis validated that follicular keratinocytes were most likely to signal toward differentiating monocytes (Fig. 4H), rather than established mLCs resident within the IL-34–rich interfollicular cells. Assessment of key receptor-ligand interactions identified several potential interactions via *Apoe* from follicular epithelial cells (Fig. 4I), consistent with the need for monocytes to adapt to the lipid-rich epidermal environment (61). Of the potential recruitment pathways, the Cxcl14-Cxcr4 axis was predominantly directed to monocytes, in agreement with previous work showing CXCL14-mediated recruitment of human monocytes to the epidermis before differentiation to mLCs (62). In addition, follicular epithelial cells were the exclusive source of *Jagged-1* and -*2* (*Jag1* and *Jag2*) ligands, capable of initiating Notch signaling in monocytes and, to a lesser extent, MCs (Fig. 4I and fig. S5F). We detected expression of Jag1 and Jag2 proteins on EpCAM^+^ keratinocytes, but this was not altered in the context of immune pathology (Fig. 4J and fig. S5G).

To determine whether Jagged signaling was required for differentiation of mLCs in vivo, we adapted a protocol previously used to define the role of Notch signaling in the differentiation of monocyte-derived Kupffer cells (13). Twelve days after transplant, mice received Jag2 blocking antibodies every 2 days, and epidermal myeloid cells were analyzed 3 weeks after transplant. The frequencies of CD11b^high^ monocytes entering the epidermis and EpCAM^+^ precursors were unaffected by Jagged blockade (Fig. 4K). However, we observed a variable but distinct trend toward a reduction in the generation of mLCs in the absence of Jagged signaling. Our findings therefore reveal a precise and spatially restricted hair follicle niche that permits not only recruitment but also commitment of monocytes to resident mLCs in the adult epidermis via the activation of Notch signaling. This niche is separate from the interfollicular epidermis that provides the IL-34 required for maintenance of the differentiated LC network.

### Differentiating monocytes metabolically adapt to the epidermal environment

Our data supported a scenario in which mLC differentiation was dependent on interactions at distinct epidermal sites: a hair follicle niche to recruit and potentially instruct monocyte differentiation and an interfollicular niche providing IL-34 for survival. Metabolic adaptation of macrophages to use fatty acid oxidation pathways is essential for long-term survival as quiescent TRMs (63, 64). Therefore, we postulated that only differentiated resident mLCs would show evidence of metabolic adaptation to the epidermal environment. To test this, we analyzed metabolic pathway usage across cell clusters using COMPASS (65), which identifies cellular metabolic states using scRNA-seq data and flux balance analysis (fig. S6A). These data showed that, despite being present in the epidermis during the same 3-week timeline after BMT, mLC metabolism was dominated by fatty acid oxidation, whereas MCs expressed higher levels of pathways linked to amino acid metabolism, suggesting active cellular processes. Differentiated mLCs also displayed a markedly different metabolic signature compared with Mrc1^+^ macs, despite colocalization in the epidermis. Use of SCENIC (single-cell regulatory network inference and clustering) (66) to infer transcription factor–target gene groups (regulons) that were more highly active in resident mLCs than other populations revealed that those regulons enriched within resident mLCs were dominated by transcription factors known to be up-regulated in response to hypoxia and a lipid-rich environment (*Zeb1* or *Rxra* and *Srebf2*, respectively) (fig. S6B) (67–69). These data suggested that responsiveness to the epidermal environment informs mLC development, enabling metabolic adaptation of mLCs to the lipid-rich epidermal environment.

### Notch signaling is sufficient to program mLC differentiation

**Fig. 5:**
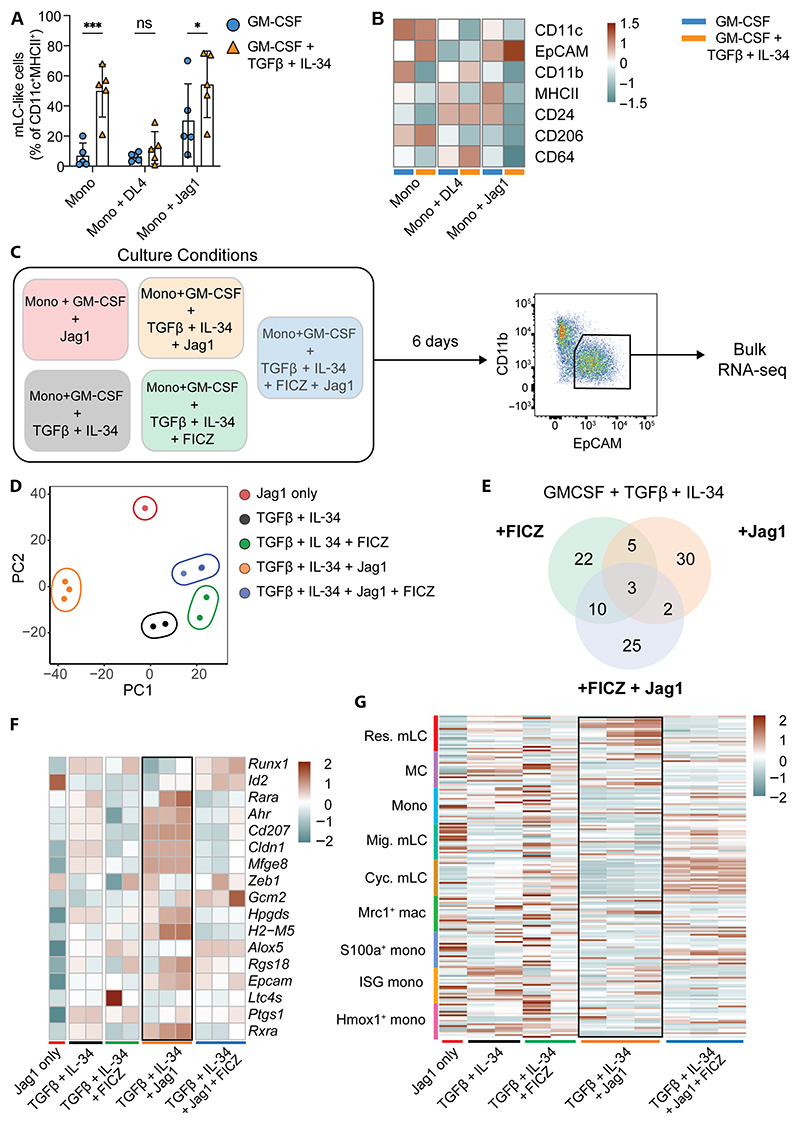
Notch signaling is sufficient to program mLC differentiation. (**A**) Bar graph showing the proportion of mLC-like cells generated from monocytes cultured with GM-CSF alone or GM-CSF, TGFβ, and IL-34 in the presence or absence of indicated Notch ligands (see fig. S7A for gating strategy). Data are shown as means ± SD (*n* = 5). Significance was calculated by two-way ANOVA with uncorrected Fisher’s least significant difference for multiple comparisons, ^*^P < 0.05; ^***^P < 0.001. (**B**) Heatmap showing average gMFI of indicated markers from BM-derived monocytes cultured and analyzed by flow cytometry as indicated in (A) (*n* = 5). (**C**) Experimental setup for bulk RNA-seq of mLC-like cells generated under the indicated conditions. (**D**) PCA plot of bulk RNA-seq samples colored by culture condition. (**E**) Venn diagram showing numbers of common and unique DEGs between the indicated conditions. (**F**) Heatmap showing scaled expression of LC signature genes across samples. (**G**) Heatmap showing expression of gene signatures from epidermal myeloid cell clusters (defined as top 20 DEGs) (*y* axis) across bulk RNA-seq samples (*x* axis).

Our data suggested a working model in which recruitment of monocytes to the upper follicular epidermis initiated a molecular cascade that resulted in loss of Zeb2 but niche-dependent activation of Notch, resulting in the differentiation of CD207^+^EpCAM^+^ *Ahr*-expressing mLCs. To define mechanistic pathways leading to an mLC fate in the adult skin, we first tested the impact of Notch signaling on monocytes. Provision of Jag1, but not the Notch ligand delta-like ligand 4 (DLL4), was sufficient to enhance differentiation of monocytes toward EpCAM^+^ mLC-like cells in vitro in the absence of TGFβ and IL-34 (Fig. 5A). We noted that DLL4 appeared to inhibit mLC development in these cultures (Fig. 5A). Although Notch signaling did not augment mLC frequencies beyond that induced by TGFβ and IL-34, we observed selection of an mLC fate at the expense of other default monocyte-derived macrophage- or DC-like cells indicated by expression of CD11c and CD206/CD64 (Fig. 5, A and B).

Therefore, to determine the effect of Notch signaling combined with other environmental signals in the skin, we cultured monocytes with GM-CSF/TGFβ/IL-34 with or without Jag1, the Ahr agonist FICZ, or both and compared the transcriptional changes within sorted CD11b^low^EPCAM^+^ mLC-like cells (Fig. 5C and fig. S7A). Cells were clustered by stimulation with considerable overlap between groups (Fig. 5, D and E). Activation of Ahr signaling did not markedly distinguish clusters along principal component 1 (PC1) and overlaid the impact of Jag1, likely because of the dominant activation of the Ahr-responsive gene *Cyp1a1* (fig. S7B and data file S1). However, provision of Jag1 signaling alone led to a marked transcriptional separation, suggesting fundamental reprogramming of EpCAM^+^ cells in this group. A comparison with our in vivo gene signatures demonstrated that Notch signaling was sufficient to program mLCs that expressed both LC-defining transcription factors (*Id2* and *Ahr*) and genes up-regulated within the skin environment (*Epcam, Cd207, Cldn1*, and *Mfge8*) (Fig. 5F, highlighted box). Moreover, a direct comparison of our scRNA-seq gene signatures defining in vivo epidermal cell populations within our bulk RNA-seq dataset demonstrated that Jag1 signaling prescribed resident mLC identity (Fig. 5G, highlighted box). The additional activation of Ahr signaling pushed cells toward a cycling mLC phenotype (Fig. 5G). Thus, Notch signaling is sufficient to restrict the differentiation potential of monocyte-derived cells, directing them away from a default macrophage program toward an mLC fate.

### Postnatal maturation of eLCs in the human skin induces expression of a DC-like immune gene program that mirrors mLC development

**Fig. 6:**
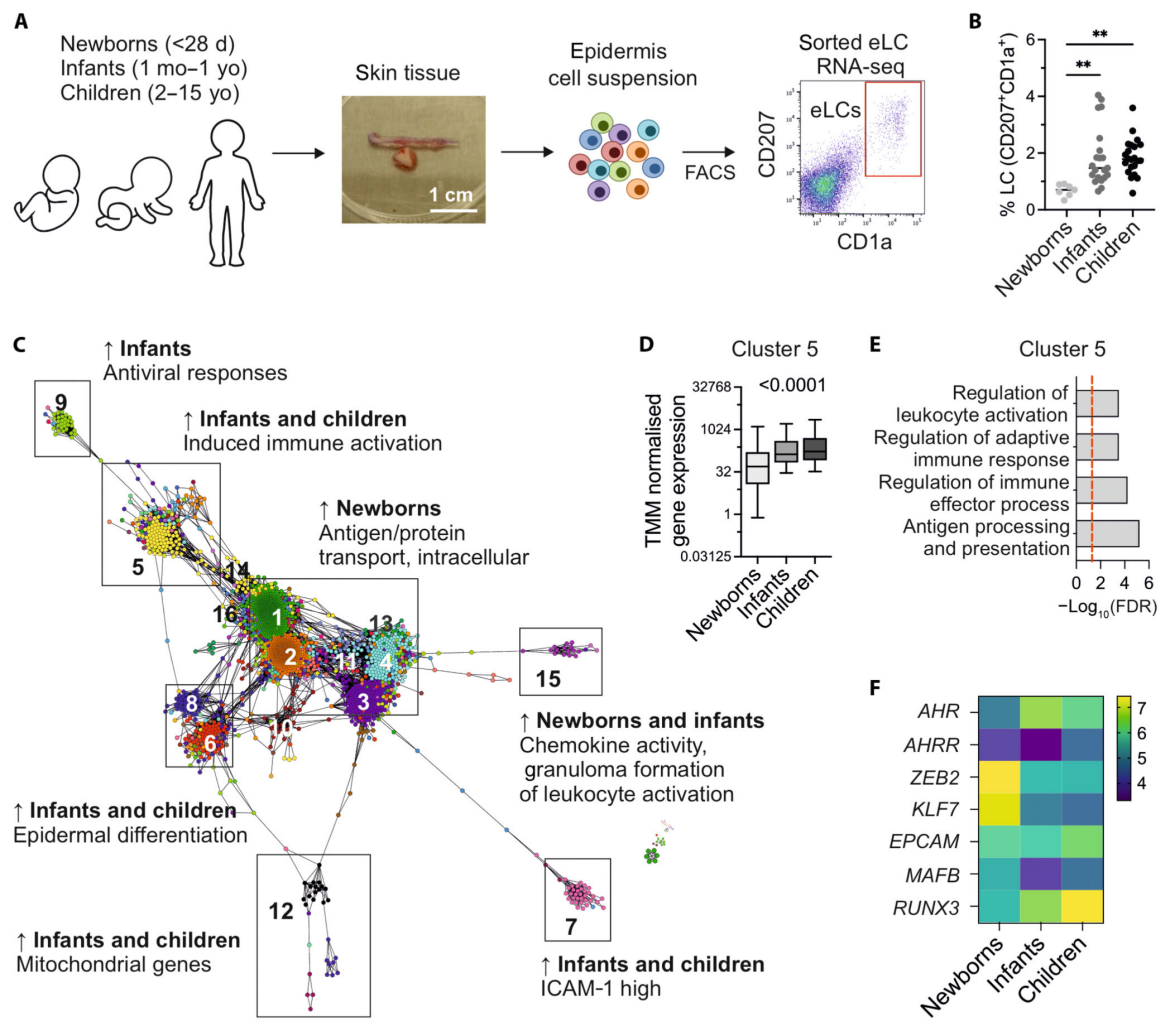
Postnatal maturation of eLCs in the human skin induces expression of DC-like immune gene programs that mirror mLC development. (**A**) Schematic showing human LC isolation workflow. Skin samples were collected from healthy donors aged 0 to 15 years old, and epidermal cell suspensions were obtained. CD207^+^CD1a^+^ cells were FACS purified directly into TRIzol. d, days; mo, months; yo, years old. (**B**) Percentage of CD207^+^CD1a^+^ cells across newborns, infants, and children. Significance was calculated by one-way ANOVA with Tukey’s multiple comparison test, ^**^P < 0.01. (**C**) Transcript to transcript clustering with visualization using Graphia, 2447 genes, *r* = 0.75, Markov Cluster (MCL) = 1.7 identified 21 clusters with *n* > 10 genes, encoding distinct transcriptional programs in human LCs. Arrows indicate enrichment. ICAM-1, intercellular adhesion molecule–1. (**D**) Average trimmed mean of M (TMM normalized gene expression levels in cluster 5 across newborns, infants, and children. Significance was calculated using one-way ANOVA. (**E**) Gene ontology ranked with FDR-corrected *P* values given for cluster 5. (**F**) Heatmap showing normalized expression of indicated genes.

Our data demonstrated that monocytes adopted a distinct pathway of differentiation in the murine adult skin, characterized by loss of *Zeb2*, to become mLCs. We therefore questioned whether such a transition away from a classical macrophage identity occurred in humans. Postnatal maturation of intestinal macrophages has been linked to the acquisition of immune functions (70). We posited that a similar maturation process occurred in the human skin after birth and was linked to LC specification. To address this, we analyzed a collection of samples taken from newborn babies (<28 days old), infants (1 month to 1 year old), and children (2 to 15 years old) by bulk RNA-seq (Fig. 6A, fig. S8A, and table S3). As previously demonstrated in mice and humans (18, 19), we observed a marked expansion in eLC numbers in the transition from newborns to infants (Fig. 6B). A visualization of the RNA-seq data as a coexpression network revealed a central gene program that is high in newborns and encodes basic macrophage functions including protein transport, RNA processing, and cadherin binding (clusters 1, 2, and 4; Fig. 6C, data file S2, and fig. S8). Specifically, there was a notable increase in transcriptional activity associated with induced immune activation from newborns to infants and children (cluster 5; Fig. 6, C and D) that was also enriched for biological processes such as antigen processing and presentation (Fig. 6E and data file S2), suggesting that more DC-like functions were activated once the skin environment and LC network have fully matured. To determine how postnatal maturation of eLCs in the human skin compared with mLC differentiation in the adult murine skin, we analyzed expression of the key factors associated with mLC development. Proliferation of eLCs in the infant skin resulted in a marked loss of *ZEB2* expression with an increase in *RUNX3*, with a trend suggesting concomitant up-regulation of *AHR* and an increase in *EPCAM* in some individuals (Fig. 6F). In summary, we demonstrate that monocytes recruited to the epidermis after immune pathology adopt a distinct pathway of differentiation to mLCs that mirrors the postnatal program in the human skin.

## Discussion

Whether LCs are macrophages or DCs has long been debated (71), and both designations are still routinely applied in the literature, despite fate-mapping studies demonstrating the embryonic macrophage origin for these cells (2, 4, 16, 72). Here, we have begun to resolve this discussion, demonstrating that differentiation within the epidermal environment drives a shift from more macrophage-like to DC-like cells. Within the skin, localization and signaling within a specialized hair follicle niche are correlated with loss of *Zeb2*, the regulator of TRM identity at other barrier sites, and Notch-dependent expression of the LC-defining transcription factors Id2 and Ahr to generate long-lived mLCs (2, 21). This adaptation to the adult skin mirrors postnatal maturation of eLCs, which is characterized by the appearance of a specific gene program associated with DC-like immune functions. Thus, specification of LCs within the skin environment drives evolution of a distinct population of TRMs, which are the only resident macrophages that can migrate to draining LNs and prime T cell immunity.

There is a growing awareness of the heterogeneity within Ly6C^+^ monocytes that represents a pool of cells derived from either GMPs or MDPs (33–35). By exploiting the Ms4a3-reporter mouse, we have tracked the fate of these ontogenically distinct monocytes in the epidermis. GMP-Mos are more common in the blood and have been shown to preferentially seed the lungs upon viral infection (35). Consistent with this, most monocytes in the epidermis were Ms4a3-labeled GMP-Mos. However, we show that MDP-Mos also contribute to the epidermal pool and that both GMP-Mos and MDP-Mos appear to differentiate via a common intermediary to become mLCs. Further studies are needed to determine whether these intrinsic differences may be linked to the LC populations identified in the healthy human skin (39) and potentially translate to differential LC function.

Evidence for the macrophage origin of embryonic LCs comes from fate-mapping studies in which labeled yolk sac (2, 72) and fetal liver (16) macrophage/monocytes gave rise to the nascent LC network in the developing embryo. Moreover, use of the macrophage-restricting gene *Mafb* to lineage trace myeloid cells labeled all LCs in the healthy adult skin (73), and generation of LCs from CD34^+^ stem cells requires suppression of the transcription factor Klf4 associated with differentiation of moDCs (74). However, LCs express high levels of CD24 and Zbtb46, considered markers of conventional DCs (73). Moreover, activation and migration initiate the expression of a convergent transcriptional program shared with DC populations leaving the skin (40, 75, 76). Migrating LCs up-regulate an interferon regulatory factor 4 (IRF4)–dependent gene program that is also evident in moDCs (38, 50). We believe that our data begin to reconcile this dichotomy. Zeb2 is a critical regulator of cell fate that specifies TRM identity across barrier sites (11, 12), and we show that only those cells that have lost *Zeb2* expression up-regulate *EpCAM* to become mLCs. Key *Zeb2* regulatory elements control function in embryonic and hematopoietic stem cell–derived macrophages (77), and molecular cross-talk with *Id2* has been shown to determine conventional (c)DC2 specification (78). We speculate that a similar process may permit expression of Id2 in differentiating mLCs and initiating the shift from more macrophage-like to DC-like cells. Further studies are needed to dissect the potential molecular interactions between *Zeb2* and *Epcam* and/or other LC-defining genes.

To determine the extrinsic signals regulating mLC development, we characterized the epidermal niche and identified a spatially restricted area of the follicular epidermis at which homotypic binding of EpCAM^+^ MCs provides access to local Notch signals. The epidermis is composed of layers of stratified epithelial cells, which are interspersed in the nonglabrous skin with follicular structures that support the cycles of hair growth. Within follicular epithelial cells, tightly demarcated areas of CCL2 expression at the upper follicular isthmus are associated with recruitment of monocytes to the epidermis (56). In addition, spatially distinct sites of integrin expression regulate activation of latent TGFβ, with α_v_β8 required for accumulation of LCs around the isthmus region and α_v_β6 needed to establish the mature LC network in the interfollicular epidermis (59). MHCII^+^CD11b^+^ cells accumulated around hair follicles upon induction of inflammation in LC-depleted BM chimeras, and LC repopulation was impaired in mice lacking hair follicles (56). However, whether the hair follicle site provides a differentiation niche or merely serves as a point of entry for monocytes into the epidermis was not known. Our data suggest that the follicular niche not only is a site of recruitment but also provides instructive signals via Notch signaling for differentiation to mLCs before they relocate within the keratinocytes of the differentiated epidermis. This finding may explain the inefficiency with which recruited epidermal monocytes were predicted to become long-lived mLCs (32), given that LC precursors will compete for Notch signals within a spatially restricted hair follicle niche. Our findings reflect the importance of Notch for differentiation of monocyte-derived Kupffer cells in the liver (13), hinting at shared exploitation of Notch pathways across tissue macrophage niches, although we identified Jagged as the ligand rather than DLL4 in the liver (13). However, we suggest that the physical restraints in the epidermis and separation from the circulation impose a two-niche model whereby hair follicle keratinocytes instruct LC molecular identity, but the differentiated intrafollicular keratinocytes provide the scaffold and trophic factors that support survival and permit adaptation to the lipid-rich epidermal environment.

Notch signaling has previously been linked to human mLC development in vitro (22, 79, 80), whereas a recent study that used scRNA-seq of human LCs revealed the presence of two eLC populations in the skin, linking Notch signaling to expansion of EpCAM^neg^ cells (39). By contrast, our data suggest that Notch signaling restricts murine monocyte differentiation into mLCs at the expense of other fates. It is possible that Notch signaling promotes a more DC-like program in mLCs. Notch2 signaling has been shown to promote differentiation and function of cDC2s in a variety of tissues (81–83). Furthermore, Notch signaling in monocytes has been shown to suppress a macrophage fate in favor of a DC fate and is required for differentiation to monocyte-derived CD207^+^CD1a^+^ cells characteristic of LC histiocytosis (84). In addition, activation of Notch in human CD1c^+^ DCs is sufficient to promote differentiation to LC-like cells that contain Birbeck granules (85).

Ahr is an evolutionarily conserved cytosolic sensor that functions as a ligand-dependent transcription factor to control cell fate decisions in gut immune cells (86) and direct monocytes toward a DC-like rather than a macrophage fate (52). The role of Ahr signaling in LCs remains unclear; eLCs begin to express Ahr upon differentiation in utero (2), but our data suggest that expression increases after birth. The epidermis of Ahr-deficient mice is replete with LCs (53), albeit a less-activated population, probably because of the absence of dendritic epidermal T cells and reduced GM-CSF production in the skin of these mice (87). Moreover, mice fed with chow deficient in Ahr dietary ligands had normal numbers of LCs (88), but these cells did not migrate to draining LNs. In contrast, LC-specific deletion of Ahr led to a reduction in epidermal LCs (89). We tested the role of Ahr signaling for the differentiation of mLCs and found that blockade of Ahr prevented monocyte differentiation in vitro, supporting a previous study using CD34^+^ precursors (90).

However, use of competitive chimeras demonstrated that Ahr-deficient monocytes could become mLCs in vivo. We observed that canonical (*Cyp1b1*) Ahr signaling was active in mLCs in vitro but not in vivo, suggesting activation of alternative pathways within the epidermal environment.

Murine and human eLCs undergo a burst of proliferation after birth (18, 19), but whether expansion of eLCs was associated with maturation of the network, as has been demonstrated for the LCs of the oral mucosa (91), was unknown. To address this question, we assembled an RNA-seq dataset from eLCs sorted from newborn children, up to 1-year-old infants, and older children. These data revealed the documented increase in LC density after the first 28 days of life (19) and demonstrated that this increase was associated with a marked change in gene expression whereby the macrophage-associated genes *MAFB* and *ZEB2* were down-regulated, whereas we observed trends toward an increase in *AHR* and *EPCAM*. This transition was accompanied by the expression of gene modules associated with enhanced immune and DC-like functions. These findings support our proposed concept of gene regulatory networks defining LC function whereby interaction between Ahr and Irf4 activates expression of immunogenic function and migration to LNs (76). The signals that trigger eLC proliferation in the skin are not known. Although skin commensal bacteria per se are not required for LC development and survival (92), it is possible that the increase in microbiota diversity during the first year of life could play an important role in conditioning the LC niche, although further experiments are required to test this hypothesis. In conclusion, convergent evolution of monocytes within the adult skin imposes expression of these gene programs to mirror postnatal conditioning and to maintain this distinct population of DC-like cells in the epidermis.

## Materials and Methods

### Study design

The aim of this study was to define intrinsic and extrinsic factors that shaped repopulation of epidermal LCs with monocytes in the inflamed skin. We used an in vivo model of LC replacement and measured changes in cell populations by combining scRNA-seq with flow cytometry and confocal microscopy. Sample sizes were based on previous experiments and the availability of genetically engineered donors. No outliers were excluded, and the numbers of replicates and independent experiments are given in each figure. The nature of our model means that we transplanted female donor BM into male recipients, and, therefore, we were restricted by the sex of the mice used. However, for in vitro BM cultures, both males and females were used. There was no randomization, and blinding was not required for these experiments because we used objective readouts such as flow cytometry. Recipients that were cohoused were possible.

### Mice

C57BL/6 (B6) mice were purchased from Charles River, UK. Langerin. DTR^GFP^ mice were originally provided by A. Kissenpfennig and B. Malissen (Centre d’Immunologie de Marseille-Luminy, CNRS, Marseille, France) (93), T cell receptor transgenic anti-HY Matahari mice were provided by J. Chai (Imperial College London, London, UK) (36), and CD45.1 mice were bred in house at University College London (UCL) Biological Services Unit. ID2^BFP^ reporter mice were a gift from A. McKenzie (University of Cambridge). The pBAD-mTagBFP2 plasmid was a gift from V. Verkhusha (Albert Einstein College of Medicine, NY, US) (Addgene plasmid no. 34632, http://n2t.net/addgene:34632, RRID:Addgene_34632) (55, 94). All procedures were conducted in accordance with the UK Home Office Animals (Scientific Procedures) Act of 1986 and were approved by the Ethics and Welfare Committee of the Comparative Biology Unit (Hampstead Campus, UCL, London, UK).

### Human samples

Human skin samples were collected with written consent from donors with approval by the South East Coast–Brighton and Sussex Research Ethics Committee in adherence to Helsinki Guidelines (ethical approvals: REC approval: 16/LO/0999). Donor information is listed in table S3.

### BM transplants

Recipient male CD45.2 C57BL/6 mice were lethally irradiated (10.4 gray of total body irradiation, split into two doses over a 2-day period) and reconstituted 4 hours after the second dose with 5 × 10^6^ female CD45.1 C57BL/6 BM cells and 2 × 10^6^ CD4 T cells, with 1 × 10^6^ CD8 Matahari T cells administered by intravenous injection through the tail vein. CD4 and CD8 donor T cells were isolated from spleen and LN single-cell suspensions by magnetic activation cell sorting (Miltenyi) using CD4 (L3T4) and CD8a (Ly-2) microbeads (Miltenyi) according to the manufacturer’s instructions. In some experiments, BM from ID2^BFP^ C57BL/6 mice was used to track donor LCs. In some experiments, C57BL/6 Langerin.DTR^GFP^ male mice were used as recipients to track host LCs. To lineage trace monocyte-derived cells, BM from C57BL/6 Ms4a3^Cre/+^R26^LSL-TdTomato^:Cx3cr1^GFP/+^ female mice was used as donor cells. In some experiments, BM from Cxcr4^CreERT2^R26^LSL-TdTomato^ [Cxcr4tm1.1(cre/ERT2)Stum, donated to R.G. by R. Stumm (Jena, Germany)] was used to track donor LCs. To activate recombination, mice received three doses of 0.12 mg of tamoxifen per gram of body weight for three consecutive days in a 100-μl volume.

### Mixed chimera experiments

BM from AHR^−/−^ mice was a gift from B. Stockinger (Francis Crick Institute, London, UK) (95). Lethally irradiated male Langerin. DTR^GFP^ C57BL/6 mice received a 50:50 mix of BM from AHR-knockout and ID2^BFP^ female mice with CD4 T cells and CD8 Matahari T cells. Three weeks after transplant, epidermis and spleens were processed and analyzed for chimerism by flow cytometry.

### In vivo antibody treatment experiments

Lethally irradiated male CD45.2 C57BL/6 mice received BM from female CD45.1 C57BL/6 mice, with CD4 T cells and CD8 Matahari T cells (BMT + T cells). Mice received intraperitoneal injections of 250 μg of anti–Jag2 (1.25 mg/ml; clone HJM2-1, Bio X Cell) or anti–immunoglobulin G (IgG) isotype control (1.25 mg/ml; polyclonal; Bio X Cell) antibodies on day 12, 14, 16, and 19 after BMT + T cells. Epidermal cells were analyzed by flow cytometry on day 20 after BMT + T cells.

### Tissue processing

#### Mouse skin

Epidermal single-cell suspensions were generated as described (32, 96). Dorsal and ventral sides of the ear pinna were split using forceps. These were floated on dispase II (2.5 mg/ml; Roche) in Hanks’ balanced salt solution and 2% fetal bovine serum (FBS) for 1 hour at 37°C or overnight at 4°C, followed by mechanical dissociation of the epidermal layer by mincing with scalpels. Cells were passed sequentially through 70- and 40-μm cell strainers in 1 mM EDTA, 1% FBS, and phosphate-buffered saline (PBS) solution.

#### Human skin

Fat and lower dermis was cut away and discarded before dispase (2 U/ml; Gibco, UK) digestion for 20 hours at 4°C. Epidermal sheets were digested in Liberase (13 U/ml; Roche, UK) for 1.5 hours at 37°C.

#### BM cells

BM single-cell suspensions were prepared from femurs and tibias of donors using a mortar and pestle. Red blood cells were lysed in 1 ml of ammonium chloride (ACK buffer) for 1 min at room temperature. Cells were washed and resuspended in complete RPMI (RPMI supplemented with 10% FBS, 1% l-glutamine, and 1% penicillin-streptomycin) until used.

### In vitro cultures

#### GMP and MDP cultures

GMPs, MDPs, and Ly6C^hi^ monocytes from whole BM were fluorescence-activated cell sorting (FACS) isolated and seeded in 96-well tissue culture–treated flat-bottom plates. Cells were cultured in complete RPMI and supplemented with recombinant GM-CSF (20 ng/ml; PeproTech), TGFβ (5 ng/ml; R&D Systems), and IL-34 (8 μg/ml; R&D Systems). The medium was partially replaced on day 2 of culture and completely replaced on day 3, and cells were harvested on day 6.

#### Monocyte cultures

Monocytes were isolated from whole BM by magnetic activation cell sorting using a monocyte isolation kit (BM; 130-100-629, Miltenyi) as per the manufacturer’s instructions. Monocytes were resuspended in complete RPMI and plated at 5 × 10^5^ cells per well in tissue culture–treated 24-well plates. Cells were cultured as described above.

#### Monocyte cocultures

OP9, OP9-Jag1, and OP9-DL4 cell lines were gifted by V. Tybulewicz (Francis Crick Institute, London, UK) and were cultured in RPMI supplemented with 10% FBS, 1% l-glutamine, 1% penicillin-streptomycin, minimum essential medium non-essential amino acids (NEAA), sodium pyruvate, Hepes buffer, and β-mercaptoethanol. OP9 cells were seeded into tissue culture–treated 24-well plates at 2 × 10^4^ cells per well and incubated overnight at 37°C. The next day, after monocyte isolation, cells were counted, and 1 × 10^5^ monocytes were seeded onto OP9 cells. Cells were cultured as above.

### Flow cytometry and cell sorting

When required, cells were acquired on a BD LSRFortessa analyzer equipped with BD FACSDiva software or sorted into either complete RPMI or buffer RLT (QIAGEN) or TRIzol using a BD Aria III.

#### Mouse

Cells were distributed into 96-well V-bottom plates or FACS tubes and incubated in 2.4G2 hybridoma supernatant for 10 min at 4°C to block Fc receptors. Cells were washed with FACS buffer (2% FBS, 2 mM EDTA, and PBS) before adding antibody cocktails that were prepared in a total volume of 50 μl per test in brilliant stain buffer (BD Biosciences) and FACS buffer. Cells were incubated with antibodies for 30 min on ice and then washed with FACS buffer. Viability was assessed either by staining cells with fixable viability dye eFluor680 (eBiosciences) or propidium iodide for fixed or unfixed cells, respectively. For intracellular staining, cells were fixed and permeabilized with the Foxp3/Transcription Factor Staining Buffer Set (Invitrogen) for 30 min on ice. Cells were subsequently washed in permeabilization buffer before adding antibody cocktails that were prepared in a total volume of 50 μl per test in permeabilization buffer. Cells were incubated with antibodies for 30 min on ice and then washed with permeabilization buffer. The antibodies used are listed in table S1.

#### Human

The antibodies used for cell staining were pretitrated and used at optimal concentrations. For FACS purification, LCs were stained for CD207 (anti-CD207 PeVio700), CD1a (anti-CD1a VioBlue), and human leukocyte antigen–DR (anti–HLA-DR VioGreen, Miltenyi Biotech, UK) (table S1).

### Immunofluorescence imaging

Skin biopsies were embedded in optimal cutting temperature (OCT) compound (Leica). Sections (10 mm) were cut using a cryostat (Leica) and stored at −20°C. Tissue was blocked for 2 hours at room temperature with 5% bovine serum albumin (Sigma-Aldrich) and 5% donkey serum (Merck) in 0.01% PBS with Tween 20. Sections were incubated with primary antibodies as listed in table S2. Antibodies were detected using donkey Cy2- or Cy5-conjugated secondary Fab fragment antibodies (Jackson Laboratories), and nuclei were stained using Hoechst 33342 (1:1000; Sigma-Aldrich) and mounted using ProLong Gold antifade mounting medium. Images were acquired on a Leica SP8 confocal microscope and subsequently analyzed with National Institutes of Health ImageJ software.

### Generation of scRNA-seq data

Single-cell suspensions from murine epidermis were stained for FACS as described above. Donor or host CD11b^+^MHCII^+^ cells and CD45^neg^ cells were sorted into RPMI medium supplemented with 2% FBS and counted manually. Cell concentrations were adjusted to 500 to 1200 cells/μl and loaded at 7000 to 15,000 cells per chip position using the 10x Chromium Single Cell 5’ Library, Gel Bead, Multiplex Kit, and Chip Kit (10x Genomics, V3 barcoding chemistry) according to the manufacturer’s instructions. All subsequent steps were performed following the standard manufacturer’s instructions. Purified libraries were analyzed by an Illumina HiSeq X Ten sequencer with 150–base pair paired-end reads.

### scRNA-seq data processing and analyses

Generated scRNA-seq data were preprocessed with the kallisto and bustools workflow (97). Downstream analysis was performed with the Seurat package in R (98). Cells with <500 detected genes and >20% mitochondrial gene expression were removed from the dataset. DoubletFinder was used to identify and remove any likely doublets. These were typically less than 1% of each batch. Principal components analysis (PCA) was performed on the 2000 most variable genes, and clusters were identified using the Leiden algorithm. Clusters were annotated on the basis of the expression of key cell type–defining genes. DEGs were identified using the FindMarkers function with significance cutoffs of log_2_ fold change >2 and adjusted *P* < 0.05.

#### Enrichment scores

To calculate enrichment scores for specific gene signatures, the Seurat function AddModuleScore was used. The human migLC gene signature included 101 genes (38). The MDP-Mo and GMP-Mo signatures included 140 and 108 genes, respectively (35).

#### Trajectory analyses

Pseudotime trajectory inference of differentiation and RNA velocity analysis based on spliced and unspliced transcript ratios were performed using the Slingshot (45) and velociraptor packages for R (44), respectively. Expression of genes changing along the trajectories was identified with general additive models fitted by tradeseq.

#### Receptor-ligand interaction analysis

Potential receptor-ligand interactions between the follicular keratinocyte subset and monocyte-derived cell clusters were investigated using the LIANA package (60). LIANA is an umbrella framework that creates a consensus receptor-ligand score from the methods and pathway tools of several other software packages. It encompasses CellPhoneDB (v2), CellChat, NATMI, iTALK, and CytoTalk (60).

#### SCENIC analysis

SCENIC was used to identify regulons, sets of transcription factors, and their cofactors coexpressed with their downstream targets in single-cell data (66). This analysis was applied to the 10x scRNA-seq dataset, and each of the regulon area under the curve per cell scores was used to identify regulons with the greatest mean difference between resident mLCs and all other clusters.

#### COMPASS analysis

In silico flux balance analysis was conducted via COMPASS (65). Normalized scRNA-seq counts per million of gene expression profiles were exported, and COMPASS analysis was conducted using standard settings on a high-performance computing cluster (99). Metabolic reactions were mapped to RECON2 reaction metadata (100), and reaction activity scores were calculated from reaction penalties. Reactions that do not have an enzyme commission number or for which there is no biochemical support (RECON2 confidence score = 1 to 3) were excluded from the analysis. Differential reaction activities were analyzed via Wilcoxon rank sum testing, and resulting *P* values were adjusted via the Benjamini-Hochberg method. Reactions with an adjusted *P* value of less than 0.1 were considered differentially active. Effect sizes were assessed with Cohen’s *d* statistic.

### Bulk RNA-seq and analyses

#### Mouse

Up to 100,000 cells were FACS sorted into RLT lysis buffer (QIAGEN) supplemented with 14 mM β-mercaptoethanol. Cells were vortexed immediately after being sorted to ensure cell lysis. RNA was extracted using the RNeasy Micro kit (QIAGEN) as per the manufacturer’s instructions, with an additional DNA clean-up step using ribonuclease-free deoxyribonuclease I (QIAGEN). RNA quantification and quality check were carried out by Novogene (UK), as well as subsequent library preparation and sequencing. Sequencing was performed on a NovaSeq 600 System (Illumina) to yield an average of 30 million reads per sample. RNA-seq transcript abundance was quantified using the salmon read mapper and an Ensembl GRCm39 transcript model. The data were imported to the R statistical environment and summarized at the gene level (that is, transcript counts summed) with tximport. Statistical transformations for visualization (vst and log_10_) and analyses of differential expression were performed using the DESeq2 package (101). Multiple testing adjustments of differential expression used the Benjamini-Hochberg false discovery rate (FDR).

#### Human

RNA was isolated using a Direct-zol RNA micro prep kit (Zymo, UK) as per the manufacturer’s protocol. RNA concentration and integrity were determined with an Agilent Bioanalyzer (Agilent Technologies, Santa Clara, CA). Preparation of RNA-seq libraries and sequencing were carried out by Oxford Genomics Centre, UK. cDNA libraries were generated using SMART-Seq Stranded Library Preparation for Ultra Low Input according to the SMART-Seq Stranded Kit User Manual following the Ultra Low Input workflow (Takara Bio). Samples were pooled (12 per batch) for library preparation. Amplified libraries were validated on the Agilent Bioanalyzer 2100 to check the size distribution and on the Qubit High Sensitivity to check the concentration of the libraries. All of the libraries passed the quality check step. Sequencing was done on an Illumina HiSeq 4000 instrument, with 150–base pair paired-end runs and 20 × 10^6^ reads per sample.

### Reverse transcription PCR

RNA was extracted from samples as described above. RNA quantification was carried out using a Nanodrop, and cDNA was synthesized using the High-Capacity cDNA Reverse Transcription kit (Applied Biosystems) as per the manufacturer’s instructions. Reverse transcription polymerase chain reaction (PCR) was run on a QuantStudio 5 Real-Time PCR system (Thermo Fisher Scientific) using Maxima SYBR Green/ROX qPCR Master Mix (2×) (Thermo Fisher Scientific) according to the manufacturer’s instructions. Primers used in this study were as follows: Ahr, AGCCGGTGCAGAAAACAGTAA (forward) and AGGCGGTCTAACTCTGTGTTC (reverse); Cyp1b1, ACGACGATGCGGAGTTCCTA (forward) and CGGGTTGGGAAATAGCTGC (reverse); and glyceraldehyde-3-phosphate dehydrogenase (GAPDH), CGGGTTCCTATAAATACGGACTGC (forward) and GTTCACACCGACCTTCACCA (reverse).

### Statistical analyses

All data, apart from RNA-seq data, were analyzed using GraphPad Prism Version 6.00 for Mac OsX (GraphPad Software, US). All line graphs and bar charts are shown as means ± SD. Protein expression data for flow cytometry are shown as geometric mean fluorescent intensity (as specified in figure legends) with the range. Significant differences were determined using one-way analysis of variance (ANOVA) to measure a single variable in three groups or two-way ANOVA for experiments with more than one variable, with post-tests specified in individual figure captions. For comparisons between two paired groups, a paired *t* test was used according to a normality test. Significance was defined as ^*^P < 0.05, ^**^P < 0.01, ^***^P < 0.001, and ^****^P < 0.0001. Statistical details of the data can be found in each figure caption. Analysis of bulk and scRNA-seq data was performed in the R and Python environments using tests described in the method details (namely, the “scRNA-seq data processing and analyses,” “COMPASS analysis,” and “Bulk RNA-seq analyses” sections).

## Supplementary Material

Fig S1-8

## Data Availability

The sequencing data for this study have been deposited in the geo genomics data repository database and can be found as NCBI GEO GSE247878 (murine scRNA-seq epidermal CD11b^+^MHCII^+^ cells), GSE247874 (murine bulk RNA-seq monocyte-derived EpCAM^+^ cells), and gSe251705 (human bulk RNA-seq CD207^+^CD1a^+^ LCs). tabulated data underlying the figures are provided in data file S3. All other data needed to support the conclusions of the paper are present in the paper or the Supplementary materials.
