# Supplementary material for: Convergent evolution of monocyte differentiation in adult skin instructs Langerhans cell identity
Source: Sci Immunol. Author manuscript; Available in PMC 2024 Oct 24. (PMC7616733; doi:10.1126/sciimmunol.adp0344)
Supplement: Fig S1-8 [file EMS199473-supplement-Fig_S1_8.pdf]

Supplemental Information

Convergent evolution of monocyte differentiation in adult skin instructs Langerhans cell identity

Anna Appios, James Davies, Sofia Sirvent, Stephen Henderson, Sébastien Trzebanski, Johannes Schroth, Morven L. Law, Inês Boal Carvalho, Marlene Magalhaes Pinto, Cyril Carvalho, Howard Yuan-Hao Kan, Shreya Lovlekar, Christina Major, Andres Vallejo, Nigel J. Hall, Michael Ardern-Jones, Zhaoyuan Liu, Florent Ginhoux, Sian M. Henson, Rebecca Gentek, Elaine Emmerson, Steffen Jung, Marta E. Polak and Clare L. Bennett.

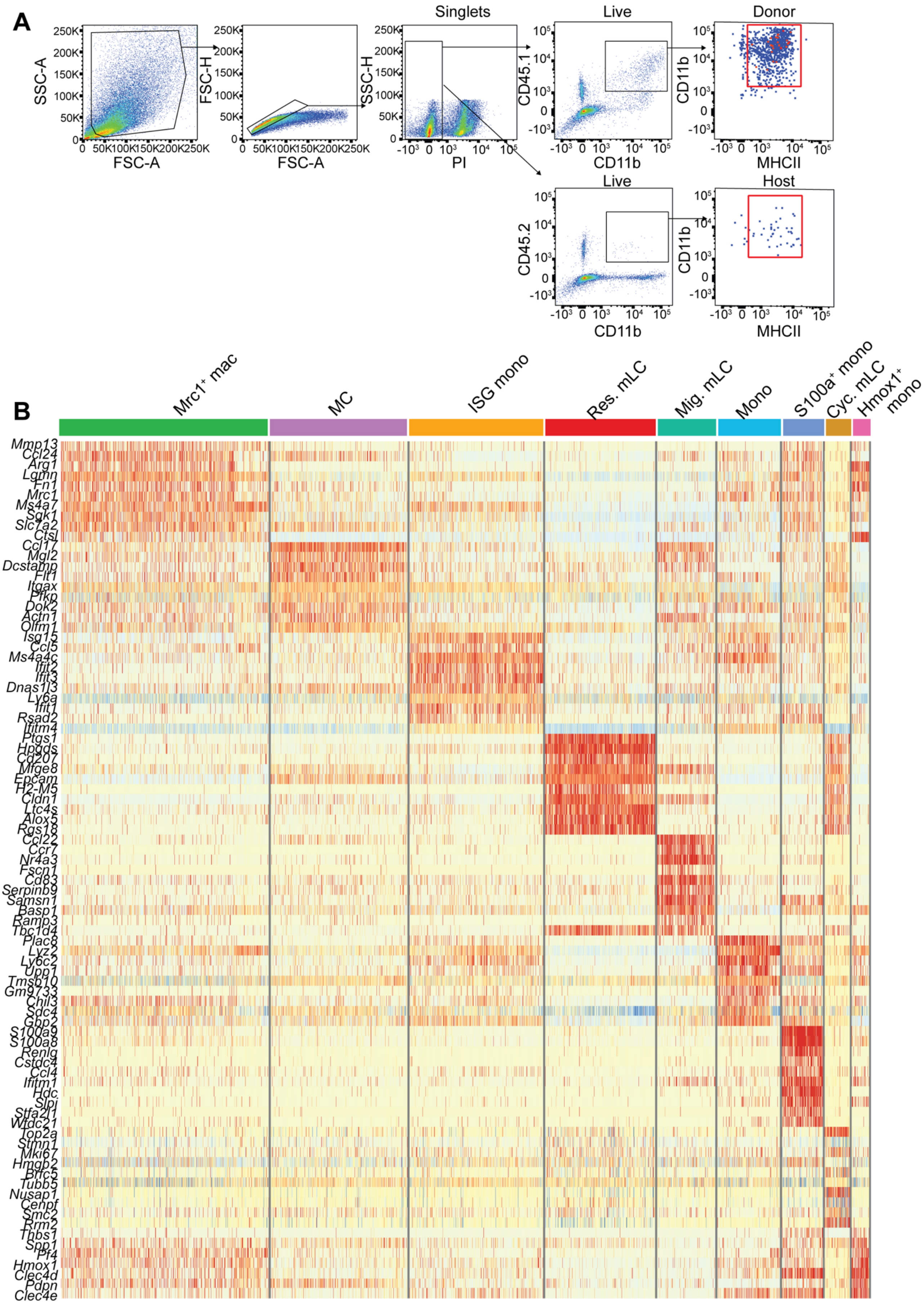
A
SSC-A
FSC-A
FSC-H
SSC-H
PI
Singlets
Live
CD45.1
CD11b
Donor
MHCII
CD45.2
Host
B
Mrc1⁺ mac
MC
ISG mono
Res. mLC
Mig. mLC
Mono
S100a⁺ mono
Cyc. mLC
Hmox1⁺ mono
Mmp13
Ccl24
Arg1
Lgmn
Fn1
Mrc1
Ms4a7
Sgk1
Slc7a2
Ctsl
Ccl17
Mgl2
Dcstamp
Flt1
Itgax
Pfkp
Dok2
Actn1
Olfm1
Isg15
Ccl5
Ms4a4c
Ifit2
Ifit3
Dnase1l3
Ly6a
Ifit1
Rsad2
Ifitm4
Ptgs1
Hpgds
Cd207
Mfge8
Epcam
H2-M5
Cldn1
Ltc4s
Alox5
Rgs18
Ccl22
Ccr7
Nr4a3
Fscn1
Cd83
Serpinb9
Samsn1
Basp1
Ramp3
Tbc1d4
Plac8
Lyz2
Ly6c2
Upp1
Tmsb10
Gm9733
Chil3
Sdc4
Gbp2
S100a9
S100a8
Retnlg
Cstdc4
Ccl4
Ifitm1
Hdc
Slpi
Stfa2l1
Wfdc21
Top2a
Stmn1
Mki67
Hmgb2
Birc5
Tubb5
Nusap1
Cenpf
Smc2
Rrm2
Thbs1
Spp1
Pf4
Hmox1
Clec4d
Pdpn
Clec4e

Figure S1. scRNA-seq of monocyte-derived cells isolated from the epidermis 3 weeks post-BMT with T cells, related to Figure 1. A. Gating strategy for FACS isolation of donor and host $CD11b^+MHCII^+$ cells from murine GVHD epidermis for 10X scRNA-seq. **B.** Heatmap showing differentially expressed genes across clusters from dataset in figure 1B.

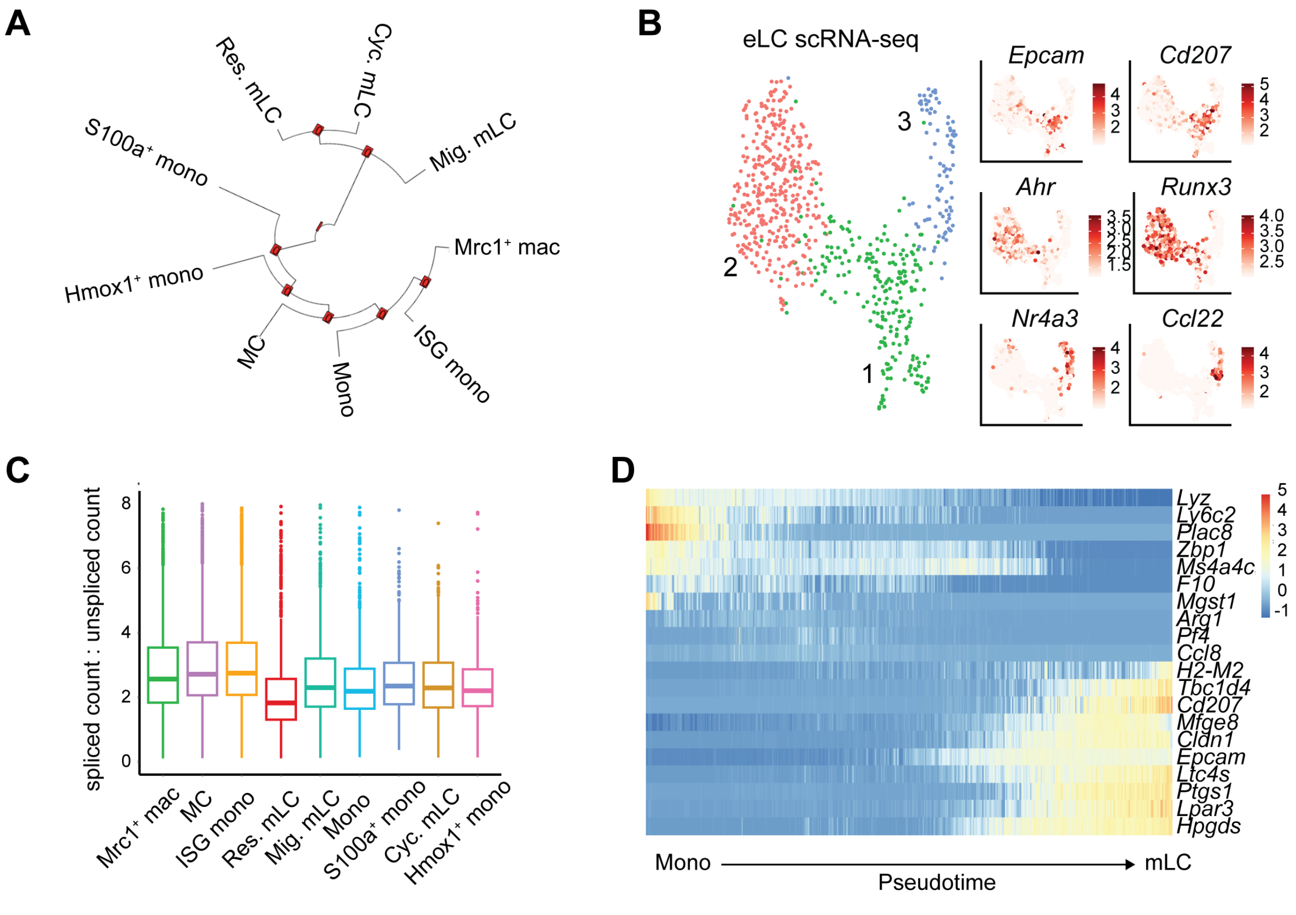

Figure S2. Analysis of scRNAseq data from epidermal myeloid cells, related to Figure 1. A. Parametric bootstrap showing single cell significance of hierarchical clustering identified in Figure 1B. **B.** UMAP and clustering of host LC (left) and heatmap overlays showing expression of indicated genes (right). **C.** Boxplot showing ratio of spliced to unspliced counts across indicated clusters. **D.** Heatmap showing the change in expression of indicated genes across pseudotime from monocyte to mLC differentiation.

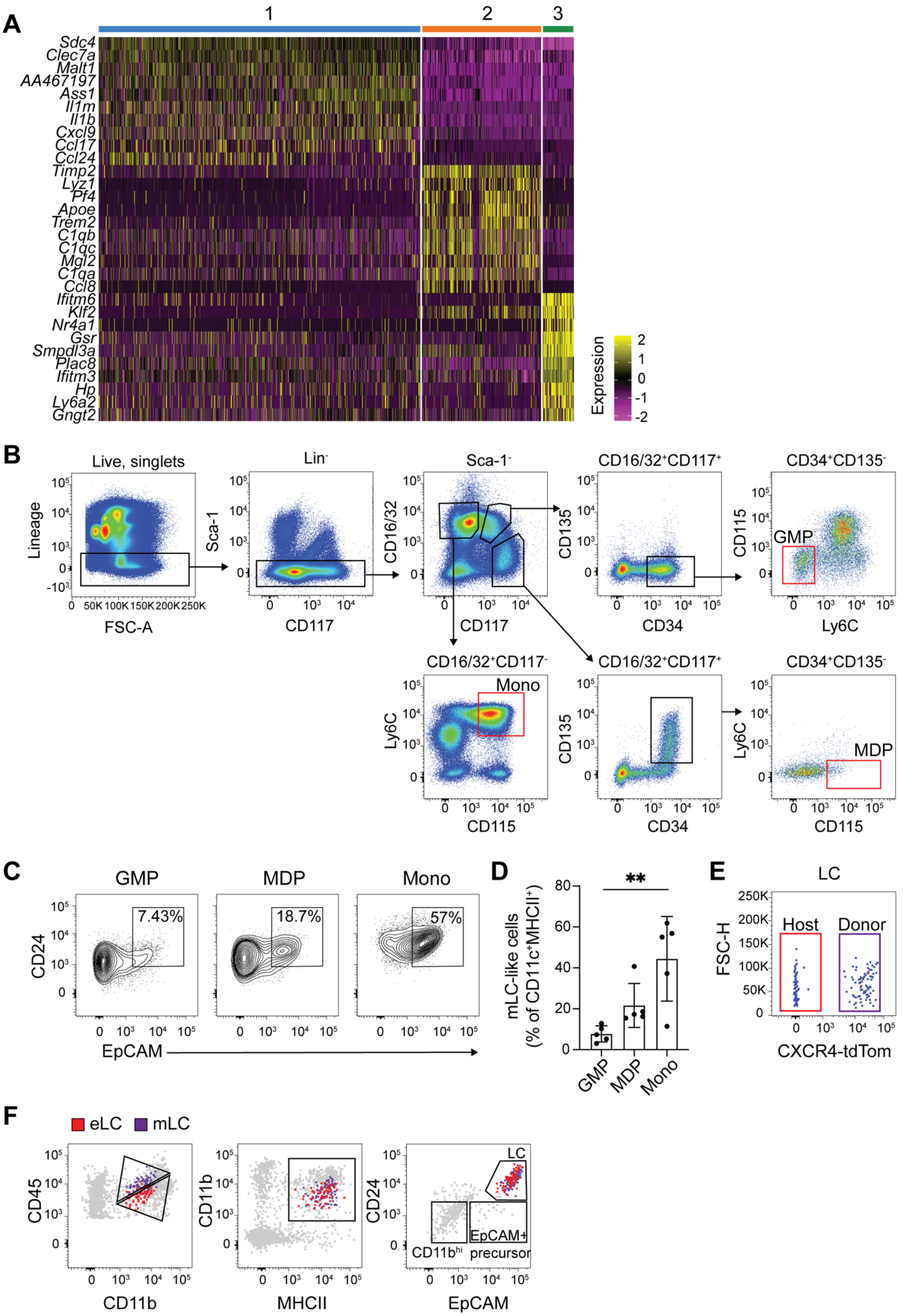
A
1
2
3
Sdc4
Clec7a
Malt1
AA467197
Ass1
Il1m
Il1b
Cxcl9
Ccl17
Ccl24
Timp2
Lyz1
Pf4
Apoe
Trem2
C1qb
C1qc
Mgl2
C1qa
Ccl8
Ifitm6
Klf2
Nr4a1
Gsr
Smpdl3a
Plac8
Ifitm3
Hp
Ly6a2
Gngt2
Expression
B
Live, singlets
Lineage
FSC-A
Lin-
Sca-1
CD117
Sca-1-
CD16/32
CD16/32+CD117+
CD135
CD34
CD34+CD135-
CD115
Ly6C
GMP
CD16/32+CD117-
Ly6C
Mono
CD115
MDP
C
GMP
MDP
Mono
CD24
EpCAM
7.43%
18.7%
57%
D
mLC-like cells
(% of CD11c+MHCII+)
**
GMP
MDP
Mono
E
LC
FSC-H
Host
Donor
CXCR4-tdTom
F
eLC
mLC
CD45
CD11b
MHCII
CD24
EpCAM
LC
CD11bhi
EpCAM+ precursor

Figure S3. Analysis of monocyte heterogeneity and differentiation to mLCs, related to Figure 2. A. Heatmap showing scaled expression of differentially expressed genes between monocyte sub-clusters. **B.** Representative FACS gating strategy for GMP, MDP and monocyte isolation (red gates) from murine bone marrow for subsequent culture. **C.** Representative flow plots showing GMP (left), MDP (middle) and monocytes (Mono, right) that were cultured for 6 days in the presence of GM-CSF, TGFβ and IL34. Gated on live, singlets, $CD45^{+}$ cells. **D.** Bar graph showing the frequency of mLC-like cells ($CD24^{+}EpCAM^{+}$) as identified in (C). Data are represented as mean±SD (n=4 independent experiments). Statistical differences were assessed using a Friedman test, ** p<0.01. **E.** Representative flow plot showing host (CXCR4-$tdTom^{neg}$) and donor (CXCR4-$tdTom^{+}$) LC identified in murine epidermis following BMT+T cells using CXCR4-tdTom donor bone marrow. In these transplants all HSC-derived donor cells will be labelled with tdTom. **F.** Cells from (E) overlaid onto the LC-defining gating strategy to show separation of $CD45^{++}$ (mLC, donor) and $CD45^{+}$ (eLC, host) LC. **G.** Representative flow plots showing donor (left) and host (right) LC identified from murine epidermis following BMT+T cells using $Ms4a3^{Cre/+}$R26LSL-^{TdTomato}:$Cx3cr1^{GFP/+}$ bone marrow.

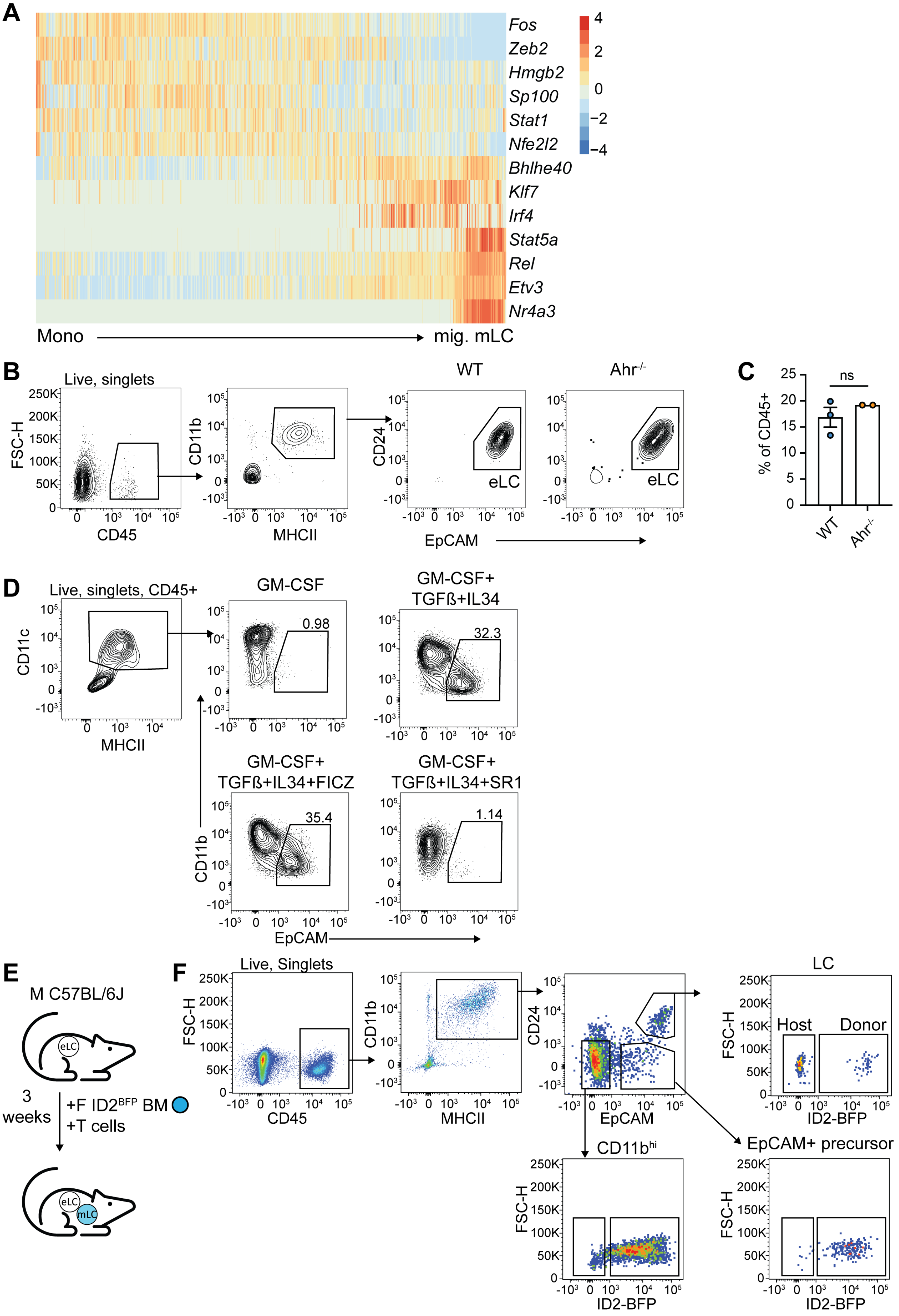
A
Fos
Zeb2
Hmgb2
Sp100
Stat1
Nfe2l2
Bhlhe40
Klf7
Irf4
Stat5a
Rel
Etv3
Nr4a3
4
2
0
−2
−4
Mono
mig. mLC
B
Live, singlets
FSC-H
CD45
CD11b
MHCII
WT
Ahr-/-
CD24
EpCAM
eLC
C
ns
% of CD45+
WT
Ahr-/-
D
Live, singlets, CD45+
CD11c
MHCII
GM-CSF
0.98
GM-CSF+
TGFß+IL34
32.3
GM-CSF+
TGFß+IL34+FICZ
35.4
GM-CSF+
TGFß+IL34+SR1
1.14
CD11b
EpCAM
E
M C57BL/6J
eLC
3
weeks
+F ID2BFP BM
+T cells
mLC
F
Live, Singlets
FSC-H
CD45
CD11b
MHCII
CD24
EpCAM
LC
Host
Donor
ID2-BFP
CD11bhi
EpCAM+ precursor

Figure S4. Transcription factors associated with mLC differentiation, related to Figure 3. A. Heatmap showing scaled gene expression of transcription factors that are differentially expressed along the differentiation trajectory (Pseudotime) from monocyte to mig. mLC. B. Gating strategy and representative flow plots showing LC from WT and $Ahr^{-/-}$ murine epidermis. C. Bar graph showing frequency of LC from WT and $Ahr^{-/-}$ epidermis. Data are represented as mean±SD (n=3 for WT, 2 for $Ahr^{-/-}$). Statistical significance was assessed using a Mann-Whitney test. D. Gating strategy and representative flow plots showing mLC-like cells generated under indicated conditions. E. Diagram of experimental set up to track in vivo mLC differentiation following GVHD. Irradiated male B6 mice receive female $ID2^{BFP}$ bone marrow, CD4 T cells with Matahari T cells and LC chimerism is assessed by flow cytometry 3 weeks later. F. Representative gating strategy used to track in vivo mLC differentiation using $ID2^{BFP}$ reporter bone marrow.

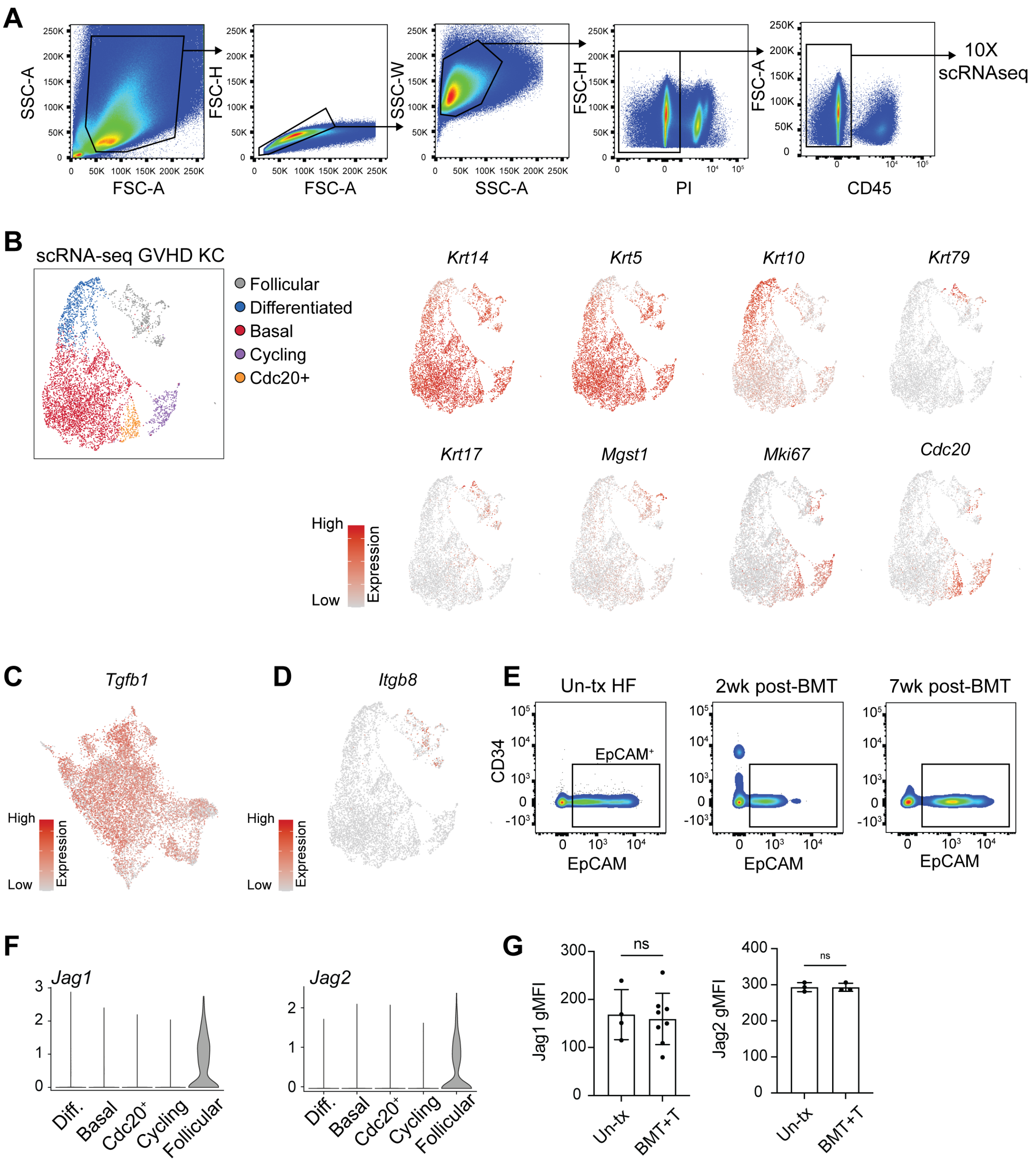

Figure S5. scRNA-seq of keratinocytes from the GVHD epidermis, related to Figure 4. A. Gating strategy for FACS isolation of keratinocytes from the GVHD epidermis for 10X scRNA-seq. **B.** UMAP visualisation and clustering of keratinocytes from GVHD epidermis analysed by scRNA-seq (left) and heatmap overlays showing expression of indicated genes across dataset (right). KC, keratinocytes. Expression scales: *Krt14* 0-6, *Krt5* 0-5, *Krt10* 0-6, *Krt79* 0-5, *Krt17* 0-6, *Mgst1* 0-5, *Mki67* 0-4, *Cdc20* 0-3. **C.** Heatmap overlay showing normalized expression of *Tgfb1* overlaid onto UMAP from figure 1B ($CD11b^{+}MHCII^{+}$ cells from GVHD epidermis). Expression scales: *Tgfb1* 0-3, *Itgb8* 0-2 **D.** Heatmap overlay showing normalized expression of *Itgb8* overlaid onto UMAP from (B). **E.** Representative flow plots showing EpCAM expression on hair follicle cells from untransplanted (Un-tx) and BMT+T epidermis. Gated on live, singlets, $CD45^{neg}$, $Sca1^{neg}$ cells. **F.** Violin plots showing normalized expression of indicated genes across scRNA-seq clusters. Diff, differentiated. **G.** Bar graphs showing gMFI of Jag1 (left) and Jag2 (right) by $EpCAM^{+}$ HF cells. Data are represented as

mean±SD (for Jag1, n=4 for Un-tx, n=8 for BMT+T cells. For Jag2, n=3 for each group). Statistical differences were assessed using a Mann-Whitney test.

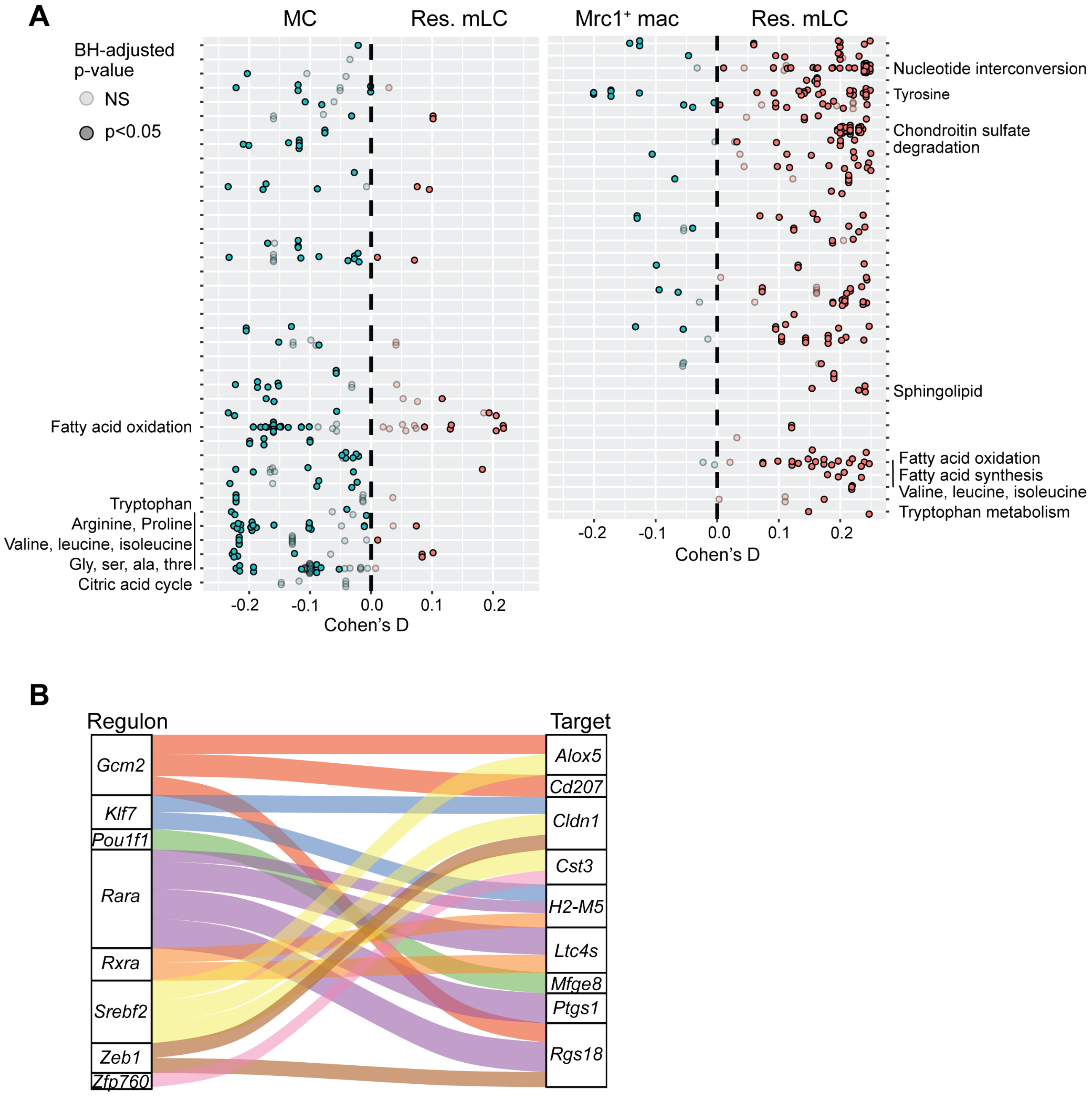

Figure S6. Metabolic adaptation of resident mLC, related to Figure 5. A. Differential activity of metabolic reactions between MC and res. mLC (left) and Mrc1^{+} mac and res. mLC (right) analysed using COMPASS. Reactions (dots) are coloured by the sign of their Cohen's D statistic, bright dots P<0.05. **B.** Relationship between 8/10 of the most enriched res. mLC regulons identified by SCENIC (Regulon; left) and 9/10 of the res. mLC signature genes shown in Fig S1B (Target; right).

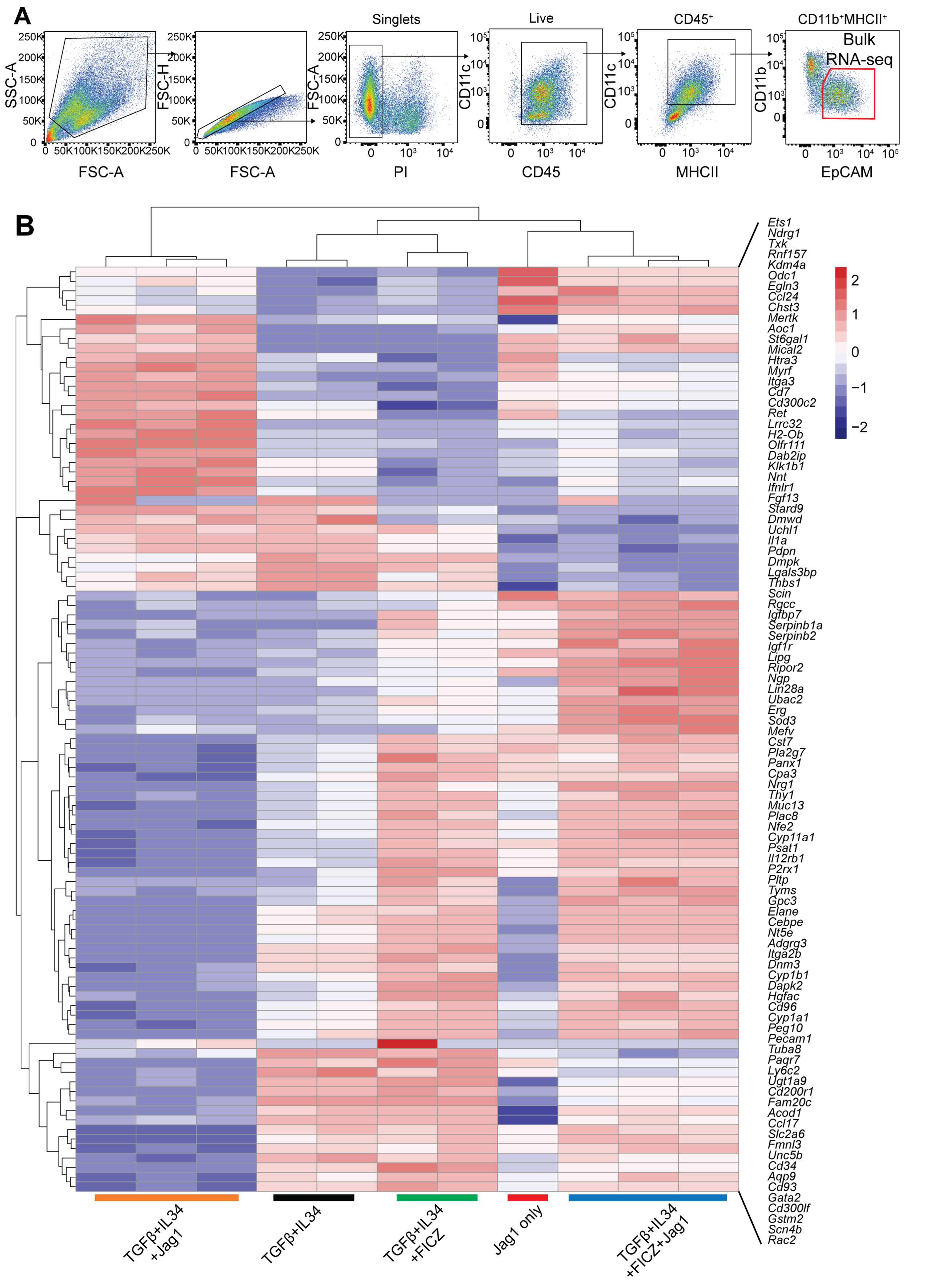

Figure S7. Bulk RNA-seq of *in vitro*-generated mLCs, related to Figure 5. A. Representative gating strategy for FACS isolation of CD11b^{int}EpCAM^{+} cells generated under conditions indicated in Figure 5C for bulk RNA-seq. **B.** Heatmap showing scaled expression of differentially expressed genes between the different indicated conditions.

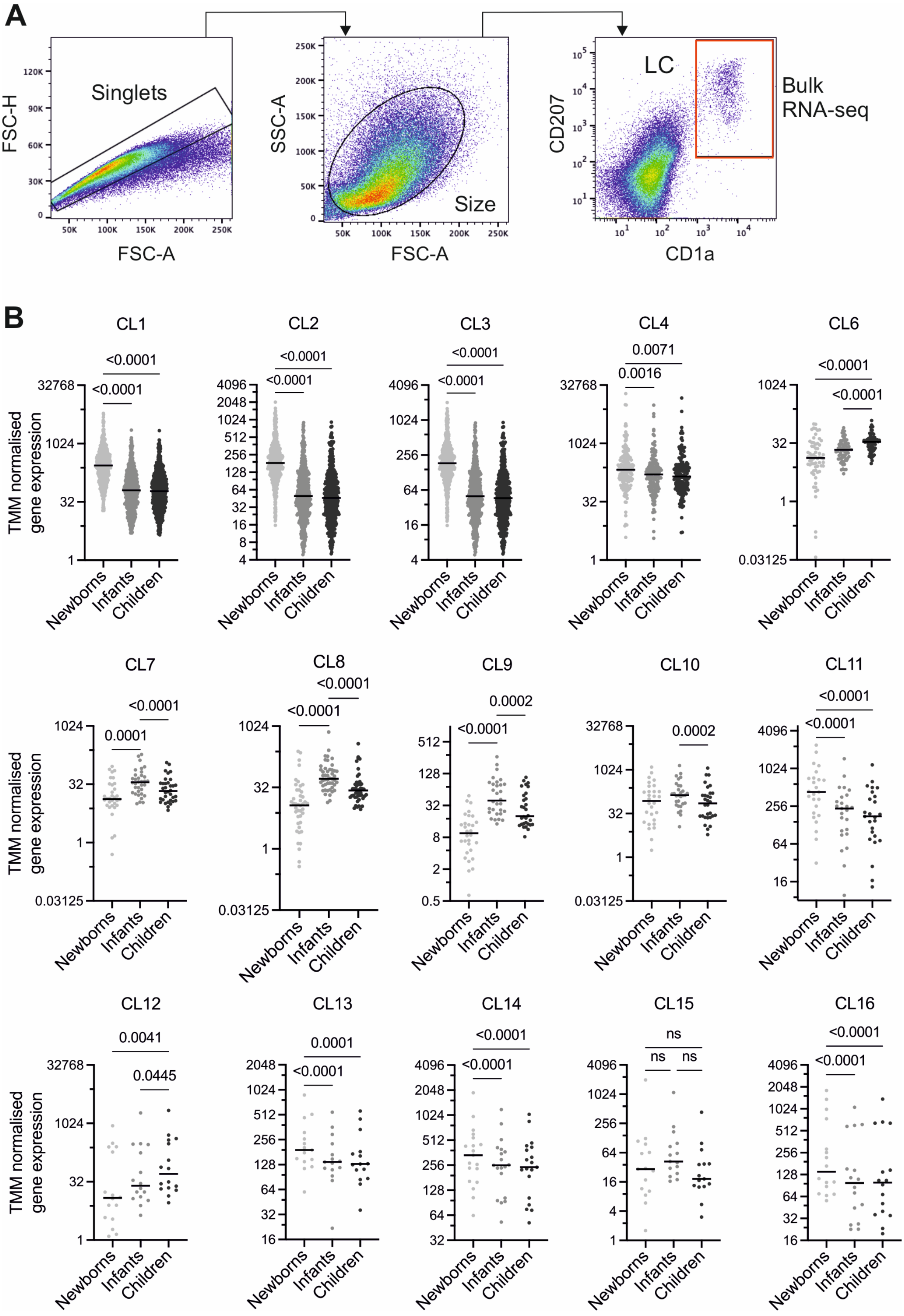

Figure S8. Bulk RNA-seq of human LCs from newborns, infants and children, related to Figure 6. A. Gating strategy for cell sorting of human epidermis samples for LC bulk RNA-seq. **B**. Average TMM normalised gene expression levels in graphia clusters across newborns, infants and children. Data are analyzed using a Two-way ANOVA with Tukey’s post-hoc test.

Antibody	Supplier	Catalog number	RRID	Dilution
**Mouse**				
Anti-mouse CD3e APC (145-2C11)	BioLegend	100312	AB_312677	1:200
Anti-mouse CD4 APC/Cy7 (GK1.5)	BioLegend	100414	AB_312699	1:200
Anti-mouse CD4 PE (GK1.5)	Thermo Fisher Scientific	12-0041-82	AB_465506	1:1000
Anti-mouse CD4 PE/Cy7 (GK1.5)	BioLegend	100422	AB_312706	1:200
Anti-mouse CD8a v450 (53-6.7)	BD Biosciences	560469	AB_1645281	1:100
Anti-mouse CD8a PE (53-6.7)	Thermo Fisher Scientific	12-0081-83	AB_465531	1:200
Anti-mouse CD11b e450 (M1/70)	Thermo Fisher Scientific	48-0112-82	AB_1582236	1:400
Anti-mouse CD11b APC (M1/70)	Thermo Fisher Scientific	17-0112-83	AB_469344	1:400
Anti-mouse CD11b PE (M1/70)	Thermo Fisher Scientific	12-0112-82	AB_2734869	1:400
Anti-mouse CD11c FITC (HL3)	BD Biosciences	553801	AB_395060	1:50
Anti-mouse CD11c APC (HL3)	BD Biosciences	550261	AB_398460	1:50
Anti-mouse CD16/32 PE-Cy7 (93)	BioLegend	101317	AB_2104156	1:200
Anti-mouse CD19 APC (1D3)	Thermo Fisher Scientific	17-0193-82	AB_1659676	1:200
Anti-mouse CD24 FITC (M1/69)	BioLegend	101805	AB_312839	1:500
Anti-mouse CD24 BV650 (M1/69)	BD Biosciences	563545	AB_2738271	1:500
Anti-mouse CD34 PE/Cy7 (SA376A4)	BioLegend	152217	AB_2910311	1:100
Anti-mouse CD34 BV421 (SA376A4)	BioLegend	152207	AB_2650766	1:100
Anti-mouse CD45 BV605 (30-F11)	BioLegend	103155	AB_2650656	1:200
Anti-mouse CD45.1 BV605 (A20)	BD Biosciences	563010	AB_2737948	1:200
Anti-mouse CD45.1 BV650 (A20)	BioLegend	110736	AB_11124743	1:200
Anti-mouse CD45.2 PerCP/Cy5.5 (104)	BD Biosciences	552950	AB_394528	1:200
Anti-mouse CD45.2 APC/Cy7 (104)	BioLegend	109824	AB_830788	1:200
Anti-mouse CD49b APC (DX5)	BioLegend	108910	AB_313416	1:200
Anti-mouse CD64 BV786 (X54-5/7.1)	BD Biosciences	741024	AB_2740644	1:100
Anti-mouse CD115 BV605 (AFS98)	BioLegend	135517	AB_2562760	1:100
Anti-mouse CD117 BV510 (2B8)	BioLegend	105839	AB_2629798	1:100
Anti-mouse CD135 PE (A2F10.1)	BioLegend	135306	AB_1877217	1:50
Anti-mouse CD206 BV711 (CO68C2)	BioLegend	141727	AB_2565822	1:100
Anti-mouse CD207 PE/Dazzle 594 (4C7)	BioLegend	144211	AB_2876491	1:200
Anti-mouse B220 BV786 (RA3-6B2)	BD Biosciences	563894	AB_2738472	1:500
Anti-mouse B220 APC (RA3-6B2)	BioLegend	103211	AB_312997	1:500
Anti-mouse EpCAM APC (G8.8)	Thermo Fisher Scientific	17-5791-82	AB_2716944	1:400
Anti-mouse EpCAM PerCP/e710 (G8.8)	Thermo Fisher Scientific	46-5791-82	AB_10598205	1:400
Anti-mouse Jagged-1 PE (HMJ1-29)	BioLegend	130907	AB_2561302	1:100
Anti-mouse Jagged-2 PE (HMJ2-1)	BioLegend	131007	AB_2128358	1:100
Anti-mouse Ly6C PE-Cy7 (AL-21)	BD Biosciences	560593	AB_1727557	1:200
Anti-mouse Ly6C FITC (AL-21)	BD Biosciences	553104	AB_394628	1:200
Anti-mouse Ly6G APC/Cy7 (1A8)	BioLegend	127624	AB_10640819	1:100
Anti-mouse Ly6G APC (1A8)	BioLegend	127613	AB_1877163	1:100
Anti-mouse MHCII BV510 (M5/114.15.2)	BioLegend	107636	AB_2561397	1:800
Anti-mouse MHCII APC/e780 (M5/114.15.2)	Thermo Fisher Scientific	47-5321-82	AB_1548783	1:800
Anti-mouse MHCII A700 (M5/114.15.2)	BioLegend	107621	AB_493727	1:400
Anti-mouse Sca-1 BV711 (D7)	BioLegend	108131	AB_2562241	1:100
Anti-mouse TER-119 APC (TER-119)	BioLegend	116212	AB_313713	1:200
Anti-mouse Vß8.3 TCR FITC (1B3.3)	BD Biosciences	553663	AB_394979	1:100
Anti-mouse Vß8.3 TCR PE (1B3.3)	BD Biosciences	553664	AB_394980	1:400
**Human**				
Anti-human CD207-PeVio700 (REA822)	Miltenyi Biotec	130-112-370	AB_2656213	1:100
Anti-human CD1a-VioBlue (REA736)	Miltenyi Biotec	130-111-875	AB_2889493	1:50
Anti human HLA-DR-VioGreen (REA805)	Miltenyi Biotec	130-111-795	AB_2652156	1:100

Table S1. List of flow cytometry antibodies used in this study.

Antibody	Supplier	Catalog number	RRID	Dilution
Anti-CD11b (M1/70)	Abcam	ab8878	AB_306831	1:200
Anti-MHCII (ER-TR3)	Abcam	ab15630	AB_302007	1:200
Anti-mouse I-A/I-E-488 (M5/114.15.2)	BioLegend	107615	AB_493524	1:200
Anti-mouse EpCAM-595 (G8.8)	BioLegend	118222	AB_2563322	1:400
Anti-KRT14 (polyclonal)	Convance	PRB-115P	AB_292096	1:500

Table S2. List of primary antibodies used for confocal microscopy in this study.
